# Sex and Etiology Differences in Overall Survival and Adverse Liver Events in Hepatocellular Carcinoma: An International Multicenter Study

**DOI:** 10.1002/mco2.71048

**Published:** 2026-11-09

**Authors:** Feng Xia, Hongwei Huang, Bin Guo, Maosen Xu, Rodrigo A. Gasque, Qian Chen, Qiao Zhang, Zhenheng Wu, Wen Chen, Zhiyuan Huang, Yiyang Liu, Liping Liu, Shuangqin Han, Songqing He, Deyu Li, Bangde Xiang, Cuncai Zhou, Wei Zhou, Yuntong Li, Shounan Lu, Chenhao Jiang, Xibin Duan, Yubo Liao, Jian Gu, Bing Zhou, Wei Cai, Jiakai Zhang, Huaxuan Yin, Guobing Xia, Yunyan Wan, Jian Li, Xiafei Xia, Huapeng Sun, Shuai Wang, Yifa Chen, Fangjun Yuan, Fanfei Lin, Wenwen Cai, Hengyi Gao, Li Ren, Xiaoping Chen, Tianyu Geng, Fabio Giannone, Carlos Moctezuma‐Velazquez, Erlei Zhang, Wellington Andraus, Qi Cheng, Ziqi Zhang

**Affiliations:** ^1^ Laboratory of Aging Research and Cancer Drug Target State Key Laboratory of Biotherapy National Clinical Research Center for Geriatrics West China Hospital Sichuan University Chengdu Sichuan China; ^2^ Department of Hepatobiliary and Pancreatic Surgery Tongji Hospital Tongji Medical College of Huazhong University of Science and Technology Wuhan Hubei China; ^3^ Hepatobiliary and Pancreatic Surgery Section General Surgery Department Clínica Universitaria Reina Fabiola Facultad De Ciencias de La Salud Universidad Católica de Córdoba Córdoba Argentina; ^4^ Department of Hepatobiliary Surgery The First Affiliated Hospital of Shihezi University Shihezi Xinjiang China; ^5^ Department of Hepatic Surgery Zhongshan People's Hospital Affiliated to Guangdong Medical University Zhongshan Guangdong China; ^6^ Department of Hepatopancreatobiliary Surgery The First Affiliated Hospital of Fujian Medical University Fuzhou Fujian China; ^7^ Department of Hepatobiliary Surgery Fuzhou First General Hospital Affiliated of Fujian Medical University Fuzhou Fujian China; ^8^ Department of General Surgery General Hospital of Central Theater Command Wuhan Hubei China; ^9^ Department of Hepatobiliary Surgery Union Hospital Tongji Medical College Huazhong University of Science and Technology Wuhan Hubei China; ^10^ Department of Hepatobiliary Surgery Shenzhen People's Hospital Shenzhen Guangdong China; ^11^ Department of Hepatobiliary Surgery Tianjin First Central Hospital Tianjin Medical University Tianjin China; ^12^ Department of Hepatobiliary Surgery The First Affiliated Hospital of Guangxi Medical University Guangxi Medical University Nanning Guangxi China; ^13^ Department of Hepatobiliary Pancreatic Surgery Henan Provincial People's Hospital Zhengzhou University Zhengzhou Henan China; ^14^ Department of Hepatobiliary Pancreatic and Splenic Surgery Guangxi Medical University Cancer Hospital Guangxi Medical University Nanning Guangxi China; ^15^ Department of Hepatobiliary Surgery Jiangxi Cancer Hospital Nanchang Medical College Nanchang Jiangxi China; ^16^ Department of Hepatobiliary Surgery Xiaogan Central Hospital Xiaogan Hubei China; ^17^ Department of Hepatobiliary and Pancreatic Surgery Zhongshan Hospital Xiamen University Xiamen Fujian China; ^18^ Department of Minimally Invasive Liver Surgery The First Affiliated Hospital of Harbin Medical University Harbin Heilongjiang China; ^19^ Department of Hepatobiliary Surgery The Second Affiliated Hospital of Army Medical University Chongqing China; ^20^ Department of Hepatobiliary and Pancreatic Surgery Zhengzhou Central Hospital Affiliated to Zhengzhou University Zhengzhou Henan China; ^21^ Department of Hepatobiliary and Pancreatic Surgery The First Affiliated Hospital of Chengdu Medical College Chengdu Sichuan China; ^22^ Department of Hepatobiliary Surgery The First Affiliated Hospital of Nanjing Medical University Nanjing Jiangsu China; ^23^ Department of General Surgery The First Affiliated Hospital of Xinxiang Medical University Xinxiang Henan China; ^24^ Department of Hepatobiliary Surgery The First Affiliated Hospital of University of Science and Technology of China Hefei Anhui China; ^25^ Department of Hepatobiliary and Pancreatic Surgery The First Affiliated Hospital of Zhengzhou University Zhengzhou Henan China; ^26^ Department of Hepatic‐biliary‐pancreatic Surgery The First People's Hospital of Foshan Foshan Guangdong China; ^27^ Department of Hepatobiliary and Pancreatic Surgery Huangshi Central Hospital Hubei Polytechnic University Huangshi Hubei China; ^28^ Department of Hepatobiliary Pancreatic Surgery Taihe Hospital Shiyan Hubei China; ^29^ Department of Hepatobiliary Surgery Renmin Hospital of Wuhan University Wuhan Hubei China; ^30^ Department of Organ Transplantation The First Affiliated Hospital of Kunming Medical University Kunming Medical University Kunming Yunnan China; ^31^ Department of General Surgery Xiangyang Central Hospital Affiliated Hospital of Hubei University of Arts and Science Xiangyang Hubei China; ^32^ Department of Hepatobiliary Surgery Jingzhou Central Hospital Jingzhou Hubei China; ^33^ Department of Hepatobiliary Surgery Ezhou Central Hospital Ezhou Hubei China; ^34^ Department of General Surgery Sinopharm Dongfeng General Hospital Hubei University of Medicine Shiyan Hubei China; ^35^ Department of Medicine Fuzhou Second General Hospital Fuzhou Fujian China; ^36^ Department of Emergency Affiliated People's Hospital of Jiangsu University Zhenjiang Jiangsu China; ^37^ Department of Hepatobiliary and Pancreatic Surgery Shenzhen Longhua District People's Hospital Shenzhen Guangdong China; ^38^ Department of Hepatobiliary Surgery Affiliated Hospital of Qinghai University Xining Qinghai China; ^39^ College of Computer Science Sichuan University Chengdu China; ^40^ Department of Hepato‐Pancreato‐Biliary Oncologic and Robotic Surgery Unit Azienda Ospedaliero‐Universitaria SS. Antonio e Biagio e Cesare Arrigo Alessandria Italy; ^41^ Department of Gastroenterology Instituto Nacional de Ciencias Médicas y Nutrición Salvador Zubirán Division of Gastroenterology (Liver Unit) University of Alberta Edmonton Canada; ^42^ Departamento De Gastroenterologia Hospital Das Clinicas HCFMUSP Faculdade De Medicina Universidade De Sao Paulo Sao Paulo Brazil

**Keywords:** adverse liver events, etiology, hepatocellular carcinoma, overall survival, sex differences

## Abstract

Sex‐associated differences in hepatocellular carcinoma (HCC) prognosis remain incompletely characterized across major etiologies. In this retrospective international multicenter cohort study, we included 7623 consecutive patients with newly diagnosed HCC from 41 centers in five countries and evaluated the associations of sex and four‐level etiology with overall survival (OS) and the first adverse liver event (hepatic decompensation, liver failure, or liver‐related death). Sex‐related OS was evaluated in the full cohort, after 1:1 propensity‐score matching (1351 men and 1351 women), and in a multivariable Cox model including recorded initial treatment modality; cause‐specific Cox and Fine–Gray models evaluated adverse liver events. Five‐year OS was 24.3% in women and 22.0% in men. Female sex was associated with lower mortality before matching (hazard ratio [HR], 0.90; 95% CI, 0.85–0.97), after matching (HR, 0.91; 95% CI, 0.84–0.99; *p* = 0.024), and after multivariable adjustment (HR, 0.91; 95% CI, 0.85–0.98; *p* = 0.010). Male sex was associated with a higher hazard of adverse liver events (cause‐specific HR, 1.26; 95% CI, 1.11–1.43; subdistribution HR [sHR], 1.23; 95% CI, 1.08–1.40). Alcohol‐related and metabolic dysfunction–associated steatotic liver disease (MASLD)‐related HCC had higher liver‐event hazards than hepatitis B virus (HBV)‐related HCC. For OS, the global sex‐by‐etiology interaction was not significant (*p* = 0.866). Female sex was associated with modestly better outcomes, although the China‐dominant cohort limits generalizability.

## Graphical abstract

Among 7623 patients with newly diagnosed hepatocellular carcinoma from 41 centers in five countries, women had modestly better overall survival and fewer adverse liver events than men. Alcohol‐related and MASLD‐related disease were associated with higher liver‐event risks than HBV‐related disease. The sex–survival association did not differ significantly across etiologies, supporting cautious, clinically contextualized interpretation in this retrospective international multicenter cohort.

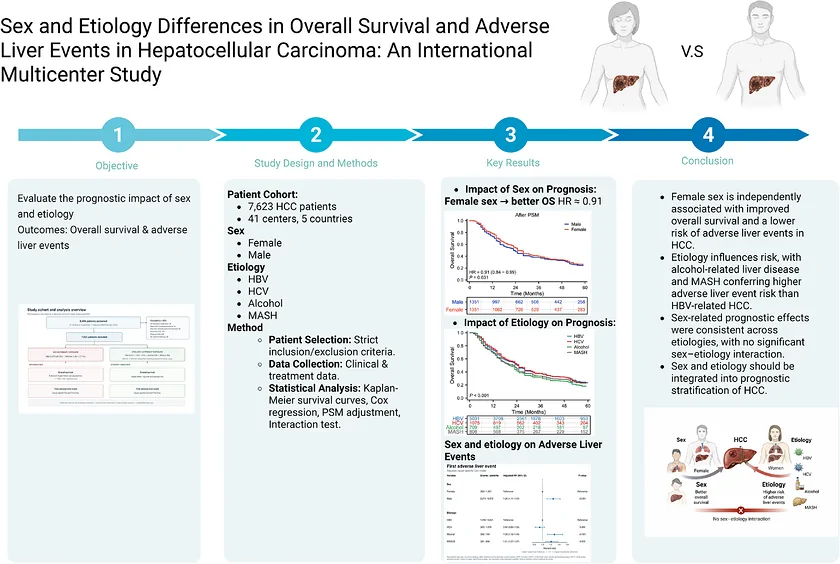

## 1 Introduction

Hepatocellular carcinoma (HCC) accounts for a considerable proportion of cancer‐related deaths worldwide [1, 2]. Despite advances in surveillance, diagnosis, and treatment, the prognosis of HCC remains highly heterogeneous, largely reflecting differences in underlying liver disease, tumor biology, and host‐related factors [3]. In addition to tumor stage and liver function, patient‐specific characteristics—including sex and disease etiology—have increasingly been recognized as important determinants of clinical outcomes in HCC.

Sex‐related disparities in HCC have been consistently observed across epidemiological and clinical studies [4, 5]. Men exhibit a substantially higher incidence of HCC than women, a phenomenon attributed to differences in sex hormones, immune responses, and exposure to etiologic risk factors [4]. Beyond incidence, accumulating evidence suggests that sex may also influence prognosis, treatment response, and survival outcomes in HCC [6, 7, 8]. Female patients have been reported to experience more favorable survival in certain clinical settings [7, 9]; however, the robustness of this survival advantage across disease stages, treatment modalities, and underlying etiologies remains incompletely understood. Etiology represents another fundamental determinant of HCC behavior and outcomes. Chronic hepatitis B virus (HBV) and hepatitis C virus (HCV) infections, alcohol‐related liver disease, and metabolic dysfunction–associated steatotic liver disease (MASLD) constitute the major causes of HCC worldwide, each characterized by distinct patterns of liver injury, tumor development, and progression [10]. Prior studies have demonstrated that etiology may influence tumor burden, liver function reserve, and long‐term prognosis [11, 12]. In particular, alcohol‐related and MASLD‐related HCC are often accompanied by advanced liver dysfunction and higher risks of hepatic decompensation, potentially leading to poorer clinical outcomes. Nevertheless, comparative data across etiological subgroups—especially in large, international cohorts—remain limited.

Importantly, sex and etiology are biologically and clinically intertwined. Differences in hormonal milieu, immune regulation, and metabolic profiles may modulate the impact of underlying liver disease on HCC progression [9, 10, 12]. However, whether the prognostic effect of sex varies according to etiology, or whether sex‐related survival differences remain consistent across etiological backgrounds, has not been systematically examined. Most existing studies are restricted by single‐center design, limited sample size, or incomplete adjustment for liver function and treatment heterogeneity, precluding definitive conclusions.

To address these gaps, we evaluated the associations of sex and four prespecified etiologic categories with overall survival and adverse liver events in a large multicenter cohort. We used propensity‐score matching, multivariable time‐to‐event models that included initial treatment, and prespecified subgroup and interaction analyses. We also assessed endpoint components, competing risks, center‐level clustering, and the influence of mixed etiologies to improve the transparency and robustness of the findings.

## 2 Results

### 2.1 Baseline Characteristics of the Study Population

Of 8444 screened patients, 821 were excluded: simultaneous malignancy (*n* = 56), mixed hepatocellular‐cholangiocarcinoma (*n* = 53), prior HCC‐directed antitumor treatment (*n* = 80), recurrent HCC (*n* = 370), ruptured HCC (*n* = 228), or unavailable outcome follow‐up (*n* = 34), leaving 7623 patients (Figure 1). Baseline characteristics of included and excluded patients are shown in Table S1, and participating centers are listed in Table S2. The country distribution was China, 7008 (91.9%); Brazil, 220 (2.9%); Argentina, 154 (2.0%); Italy, 136 (1.8%); and Mexico, 105 (1.4%) (Table S3).

**FIGURE 1:**
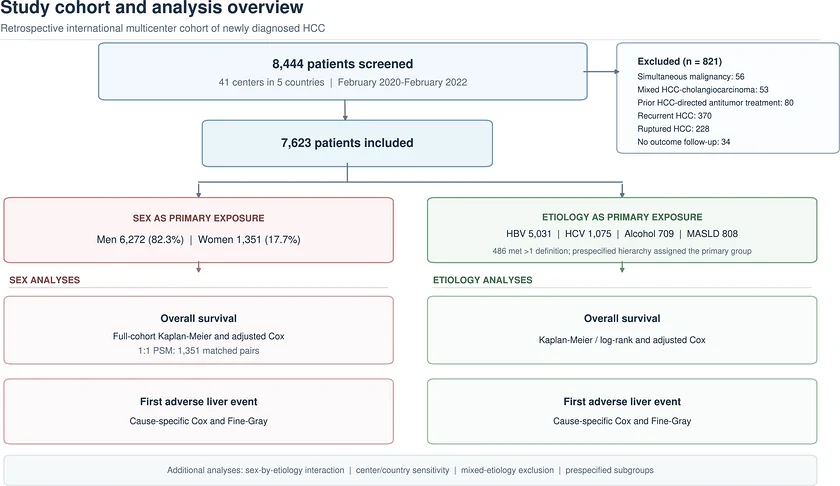
Study flow and analysis overview. After application of the six mutually exclusive exclusion categories, the full cohort contributed to analyses of both sex and etiology for overall survival and the first adverse liver event; sex‐based overall‐survival analyses additionally included 1:1 propensity‐score matching (PSM).

The cohort included 6272 men (82.3%) and 1351 women (17.7%). Etiology was HBV in 5031 patients (66.0%), HCV in 1075 (14.1%), alcohol‐related disease in 709 (9.3%), and MASLD in 808 (10.6%) (Table 1). Selected baseline characteristics by primary etiology are shown in Table S4. Four hundred eighty‐six patients (6.4%) met criteria for more than one etiologic factor; the mixed‐etiology count, deterministic assignment rule, and exclusion sensitivity analyses are reported in Table S5A,B.

**TABLE 1:** Baseline clinical characteristics of the HCC cohort (*n* = 7623).

	HCC cohort
	7623
Sex (%)	
Male	6272 (82.3)
Female	1351 (17.7)
Age (%)	
< 60 years	4611 (60.5)
≥ 60 years	3012 (39.5)
BMI (%)	
< 25 kg/m^2^	5602 (73.5)
≥ 25 kg/m^2^	2021 (26.5)
Tumor size (%)	
< 5 cm	4155 (54.5)
≥ 5 cm	3468 (45.5)
Tumor number (%)	
1	6975 (91.5)
2	445 (5.8)
≥ 3	203 (2.7)
AFP (%)	
< 400 ng/mL	3089 (40.5)
≥ 400 ng/mL	4534 (59.5)
Cirrhosis (%)	
No	1565 (20.5)
Yes	6058 (79.5)
CSPH (%)	
No	4977 (65.3)
Yes	2646 (34.7)
Child–Pugh class (%)	
A	5663 (74.3)
B	1258 (16.5)
C	702 (9.2)
ECOG (%)	
0	4993 (65.5)
≥ 1	2630 (34.5)
Primary liver disease etiology (%)	
HBV	5031 (66.0)
HCV	1075 (14.1)
Alcohol	709 (9.3)
MASLD	808 (10.6)
PVTT (%)	
No	5832 (76.5)
Yes	1791 (23.5)
History of upper abdominal surgery (%)	
No	7150 (93.8)
Yes	473 (6.2)
ALBI grade (%)	
1	6371 (83.6)
2	808 (10.6)
3	444 (5.8)
ASA (%)	
I	1638 (21.5)
II	5153 (67.6)
III	832 (10.9)
ALT (%)	
< 50 U/L	4863 (63.8)
≥ 50 U/L	2760 (36.2)
AST (%)	
< 40 U/L	3902 (51.2)
≥ 40 U/L	3721 (48.8)
ALP (%)	
< 100 U/L	5953 (78.1)
≥ 100 U/L	1670 (21.9)
GGT (%)	
< 60 U/L	5635 (73.9)
≥ 60 U/L	1988 (26.1)
Platelets (×10^9^/L)	158 (120–240)^a^
Creatinine (mg/dL)	0.87 (0.79–0.99)^a^
Bilirubin (mg/dL)	0.67 (0.60–1.00)^a^
Albumin (g/L)	43.5 (38.5–47.5)^a^
Sarcopenia (%)	
No	3926 (51.5)
Yes	3697 (48.5)
Smoking history (%)	
No	5452 (71.5)
Yes	2171 (28.5)
Drinking history (%)	
No	6174 (81.0)
Yes	1449 (19.0)
Antiviral therapy (%)	
No	2661 (34.9)
Yes	4962 (65.1)
Comorbidities	
Diabetes mellitus (%)	1350 (17.7)
Hypertension (%)	1941 (25.5)
Cardiovascular disease (%)	572 (7.5)
Chronic kidney disease (%)	396 (5.2)
COPD (%)	149 (2.0)
Recorded initial treatment modality (%)	
Surgical resection (%)	2372 (31.1)
Curative local therapy (RFA/MWA) (%)	773 (10.1)
Palliative locoregional therapy (TACE) (%)	2782 (36.5)
Systemic therapy (TKI/ICI) (%)	1368 (17.9)
Supportive care (%)	328 (4.3)

*Note*: Values are *n* (%) unless otherwise indicated. Comorbidities are not mutually exclusive, and treatment percentages may not total 100 because of rounding. For patients receiving combination treatment, recorded initial treatment modality was classified using the hierarchy surgical resection > curative local therapy (RFA/MWA) > palliative locoregional therapy (TACE) > systemic therapy (TKI/ICI) > supportive care.

^a^ Continuous variables are summarized as median (interquartile range).

Abbreviations: AFP, alpha‐fetoprotein; ALBI, albumin–bilirubin grade; ALP, alkaline phosphatase; ALT, alanine aminotransferase; ASA, American Society of Anesthesiologists score; AST, aspartate aminotransferase; BMI, body mass index; COPD, chronic obstructive pulmonary disease; CSPH, clinically significant portal hypertension; ECOG, Eastern Cooperative Oncology Group; GGT, γ‐glutamyl transpeptidase; HBV, hepatitis B virus infection; HCC, hepatocellular carcinoma; HCV, hepatitis C virus infection; ICI, immune checkpoint inhibitor; IQR, interquartile range; MASLD, metabolic dysfunction–associated steatotic liver disease; MWA, microwave ablation; PVTT, portal vein tumor thrombosis; RFA, radiofrequency ablation; TACE, transarterial chemoembolization; TKI, tyrosine kinase inhibitor.

### 2.2 Sex and Etiology Differences in Overall Survival

Before matching, cirrhosis was recorded in 5028 of 6272 men (80.2%) and 1030 of 1351 women (76.2%). Men also more frequently reported smoking and alcohol use, whereas other baseline differences were generally modest (Table S6). After 1:1 matching, each group contained 1351 patients; post‐matching characteristics are shown in Table S7. All reported absolute or generalized standardized mean differences (SMDs) were below 0.10 (maximum, 0.046; Table S8 and Figure S1).

Before matching, the estimated 1‐, 3‐, and 5‐year overall survival rates were 77.4%, 39.7%, and 24.3% in women and 72.8%, 36.1%, and 22.0% in men. Female sex was associated with lower mortality (HR, 0.90; 95% CI, 0.85–0.97; *p* = 0.003; Figure 2A). The association persisted in the matched cohort using robust sandwich variance clustered by matched pair (HR, 0.91; 95% CI, 0.84–0.99; *p* = 0.024; Figure 2B and Table S9). In the multivariable full‐cohort model that represented etiology as a four‐level variable and included recorded initial treatment, female sex remained associated with lower mortality (adjusted HR, 0.91; 95% CI, 0.85–0.98; *p* = 0.010). Alcohol‐related HCC had higher mortality than HBV‐related HCC (adjusted HR, 1.12; 95% CI, 1.02–1.23; *p* = 0.018), whereas HCV and MASLD were not significantly different from HBV (Table 2). In the inverse‐probability‐weighted analysis using stabilized weights truncated at the first and 99th percentiles, female sex remained associated with lower mortality (HR, 0.91; 95% CI, 0.85–0.98; *p* = 0.008); the Cox model stratified by matched pair gave HR 0.92 (95% CI, 0.85–1.00; *p* = 0.045; Table S9). Center‐ and country‐based sensitivity analyses, including the non‐China cohort, are summarized in Table S10 and Figure S2.

**FIGURE 2:**
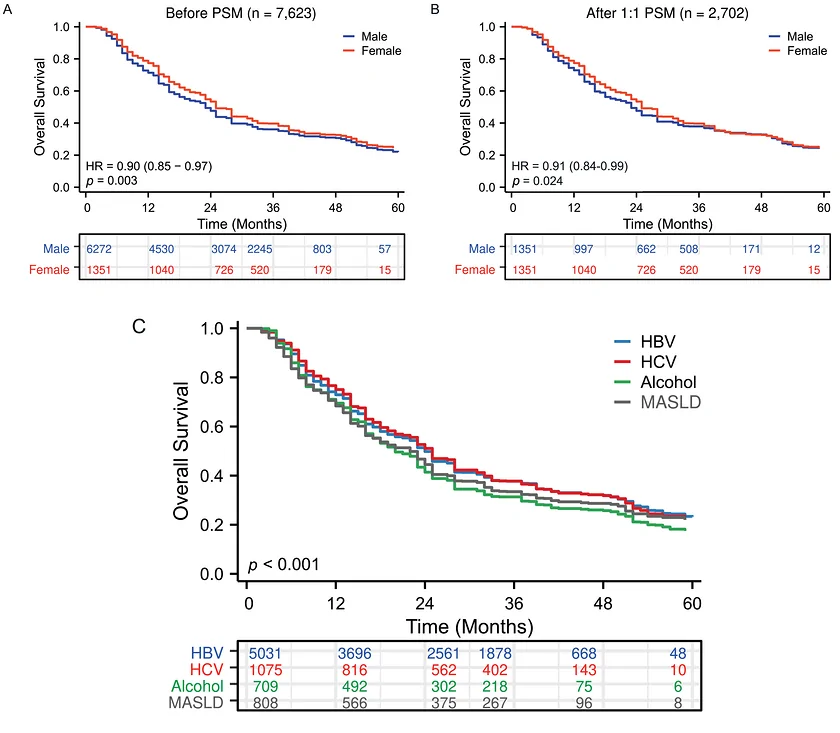
Kaplan–Meier overall‐survival curves according to sex and etiology. (A) Women versus men in the full cohort; the reported HR compares women with men. (B) Women versus men after 1:1 PSM. Because all 1351 women were retained in the matched cohort, their Kaplan–Meier curve and numbers at risk are identical in panels A and B. (C) HBV‐, HCV‐, alcohol‐, and MASLD‐related HCC in the full cohort; the displayed *p* value is from the overall log‐rank test. Numbers at risk are shown below each panel.

**TABLE 2:** Univariable and multivariable Cox proportional‐hazards analyses for overall survival.

Variable	Univariable HR (95% CI)	*p* value	Multivariable HR (95% CI)	*p* value
Sex				
Male	Reference	—	Reference	—
Female	0.90 (0.85–0.97)	0.003	0.91 (0.85–0.98)	0.010
Age, years				
< 60	Reference	—	Reference	—
≥ 60	1.06 (1.00–1.12)	0.048	1.04 (0.98–1.10)	0.201
BMI, kg/m^2^				
< 25	Reference	—	—	—
≥ 25	0.96 (0.90–1.02)	0.173	—	—
Maximum tumor diameter				
< 5 cm	Reference	—	Reference	—
≥ 5 cm	1.64 (1.54–1.75)	< 0.001	1.28 (1.19–1.38)	< 0.001
Tumor number				
1	Reference	—	Reference	—
2	1.25 (1.12–1.40)	< 0.001	1.12 (1.00–1.25)	0.050
≥ 3	1.58 (1.35–1.84)	< 0.001	1.30 (1.10–1.54)	0.002
AFP				
< 400 ng/mL	Reference	—	Reference	—
≥ 400 ng/mL	1.32 (1.24–1.41)	< 0.001	1.18 (1.10–1.26)	< 0.001
Cirrhosis				
No	Reference	—	Reference	—
Yes	1.16 (1.08–1.24)	< 0.001	1.03 (0.95–1.12)	0.460
CSPH				
No	Reference	—	Reference	—
Yes	1.35 (1.27–1.44)	< 0.001	1.12 (1.04–1.21)	0.003
Child‐Pugh class				
A	Reference	—	Reference	—
B	1.29 (1.19–1.40)	< 0.001	1.13 (1.03–1.24)	0.008
C	1.58 (1.43–1.75)	< 0.001	1.26 (1.12–1.41)	< 0.001
ECOG performance status				
0	Reference	—	Reference	—
≥ 1	1.40 (1.32–1.49)	< 0.001	1.16 (1.08–1.24)	< 0.001
Etiology (global P)		< 0.001		0.041
HBV	Reference	—	Reference	—
HCV	0.95 (0.88–1.03)	0.214	0.97 (0.89–1.06)	0.503
Alcohol	1.18 (1.08–1.29)	< 0.001	1.12 (1.02–1.23)	0.018
MASLD	1.07 (0.98–1.17)	0.129	1.05 (0.96–1.15)	0.286
Portal vein tumor thrombosis				
No	Reference	—	Reference	—
Yes	2.05 (1.91–2.19)	< 0.001	1.55 (1.44–1.67)	< 0.001
History of upper abdominal surgery				
No	Reference	—	—	—
Yes	1.05 (0.94–1.17)	0.395	—	—
ALBI grade				
1	Reference	—	Reference	—
2–3	1.42 (1.32–1.53)	< 0.001	1.17 (1.08–1.27)	< 0.001
ASA class				
I	Reference	—	—	—
II–III	1.10 (1.03–1.18)	0.006	—	—
ALT ≥ 50 U/L	1.08 (1.01–1.15)	0.021	—	—
AST ≥ 40 U/L	1.13 (1.06–1.20)	< 0.001	—	—
ALP ≥ 100 U/L	1.19 (1.11–1.28)	< 0.001	—	—
GGT ≥ 60 U/L	1.15 (1.08–1.23)	< 0.001	—	—
Platelets, per 10 × 10^9^/L	0.99 (0.98–1.00)	0.072	—	—
Creatinine, per 0.1 mg/dL	1.01 (1.00–1.02)	0.083	—	—
Bilirubin, per 1 mg/dL	1.09 (1.05–1.13)	< 0.001	—	—
Albumin, per 1 g/L	0.97 (0.96–0.98)	< 0.001	—	—
Smoking history, yes	1.08 (1.01–1.15)	0.025	—	—
Alcohol‐use history, yes	1.14 (1.05–1.24)	0.002	—	—
Antiviral therapy				
Yes	Reference	—	Reference	—
No	1.20 (1.13–1.28)	< 0.001	1.11 (1.04–1.19)	0.002
Initial treatment (global P)		< 0.001		< 0.001
Surgical resection	Reference	—	Reference	—
Curative local therapy	1.18 (1.07–1.31)	0.001	1.12 (1.01–1.25)	0.036
Palliative locoregional therapy (TACE)	1.78 (1.67–1.90)	< 0.001	1.42 (1.32–1.53)	< 0.001
Systemic therapy (TKI/ICI)	2.15 (2.00–2.32)	< 0.001	1.63 (1.50–1.77)	< 0.001
Supportive care	3.40 (3.05–3.79)	< 0.001	2.21 (1.96–2.49)	< 0.001

*Note*: Etiology is modeled as one four‐level variable with HBV as the reference. The multivariable model includes sex, age, tumor size, tumor number, AFP, cirrhosis, CSPH, Child‐Pugh class, ECOG status, etiology, portal vein tumor thrombosis, ALBI grade, antiviral therapy, and initial treatment modality. Robust standard errors are clustered by center. An em dash in the multivariable columns indicates that the variable was not entered into the prespecified multivariable model. Confidence limits and *p* values were calculated from unrounded estimates; displayed confidence limits are rounded to two decimal places. Recorded initial‐treatment coefficients are associational and should not be interpreted causally.

Abbreviations: AFP, alpha‐fetoprotein; ALBI, albumin‐bilirubin; ALP, alkaline phosphatase; ALT, alanine aminotransferase; ASA, American Society of Anesthesiologists; AST, aspartate aminotransferase; BMI, body mass index; CI, confidence interval; CSPH, clinically significant portal hypertension; ECOG, Eastern Cooperative Oncology Group; GGT, gamma‐glutamyl transferase; HBV, hepatitis B virus; HCV, hepatitis C virus; HR, hazard ratio; MASLD, metabolic dysfunction‐associated steatotic liver disease.

Overall survival differed across the four etiologic groups by the log‐rank test (*p* < 0.001), and alcohol‐related HCC had the lowest estimated 1‐, 3‐, and 5‐year survival rates (69.5%, 31.3%, and 17.5%, respectively; Figure 2C). The numbers of deaths were 3253 of 5031 in the HBV group, 691 of 1075 in the HCV group, 510 of 709 in the alcohol group, and 527 of 808 in the MASLD group (Table S11).

### 2.3 Association of Sex and Etiology With Adverse Liver Events

A first adverse liver event occurred in 393 of 1351 women (29.1%) and 2274 of 6272 men (36.3%). Male sex was associated with greater risk in the adjusted cause‐specific Cox model (HR, 1.26; 95% CI, 1.11–1.43; *p* < 0.001). Relative to HBV‐related HCC, alcohol‐related HCC (HR, 1.29; 95% CI, 1.12–1.49; *p* < 0.001) and MASLD‐related HCC (HR, 1.21; 95% CI, 1.07–1.37; *p* = 0.003) had higher risks, whereas HCV‐related HCC did not (HR, 0.93; 95% CI, 0.82–1.05; *p* = 0.252; Table 3 and Figure 3). Of 2667 first events, 1589 were hepatic decompensation (936 ascites, 381 variceal bleeding, and 272 hepatic encephalopathy), 523 were liver failure, and 555 were liver‐related death. The Fine–Gray estimate for male sex was 1.23 (95% CI, 1.08–1.40; *p* = 0.002), with concordant estimates for alcohol‐related and MASLD‐related HCC (Table S12A,B). Excluding 486 mixed‐etiology patients produced a similar estimate (male HR, 1.24; 95% CI, 1.09–1.42; *p* = 0.001; Table S5B). Center‐frailty, alternative competing‐risk, and true 90‐day landmark sensitivity analyses were directionally concordant (Table S13 and Figure S2).

**TABLE 3:** Adjusted associations of sex and etiology with the first adverse liver event.

Variable	Events/patients	Adjusted cause‐specific HR (95% CI)	*p* value	Adjusted Fine–Gray sHR (95% CI)	*p* value
Sex			< 0.001		0.002
Female	393/1351	Reference	—	Reference	—
Male	2274/6272	1.26 (1.11–1.43)	< 0.001	1.23 (1.08–1.40)	0.002
Etiology (global Wald P)			< 0.001		< 0.001
HBV	1690/5031	Reference	—	Reference	—
HCV	340/1075	0.93 (0.82–1.05)	0.252	0.95 (0.84–1.08)	0.425
Alcohol	306/709	1.29 (1.12–1.49)	< 0.001	1.25 (1.08–1.45)	0.003
MASLD	331/808	1.21 (1.07–1.37)	0.003	1.18 (1.04–1.34)	0.010

Both models were adjusted for age, sex, etiology, BMI, tumor size, tumor number, AFP, cirrhosis, CSPH, Child–Pugh class, ECOG performance status, portal vein tumor thrombosis, ALBI grade, antiviral therapy, history of upper abdominal surgery, and recorded initial treatment modality. In the cause‐specific Cox model, non‐liver‐related or indeterminate death and liver transplantation were censored at occurrence, and robust standard errors accounted for clustering by center. In the Fine–Gray model, non‐liver‐related or indeterminate death and liver transplantation were competing events, with center‐clustered robust standard errors. HRs and sHRs greater than 1 indicate a higher hazard for male versus female patients or for the listed etiology versus HBV. The Fine–Gray estimates are complementary to, rather than interchangeable with, the cause‐specific estimates.

Abbreviations: AFP, alpha‐fetoprotein; ALBI, albumin‐bilirubin; BMI, body mass index; CI, confidence interval; CSPH, clinically significant portal hypertension; ECOG, Eastern Cooperative Oncology Group; HBV, hepatitis B virus; HCV, hepatitis C virus; HR, hazard ratio; MASLD, metabolic dysfunction‐associated steatotic liver disease; sHR, subdistribution hazard ratio.

**FIGURE 3:**
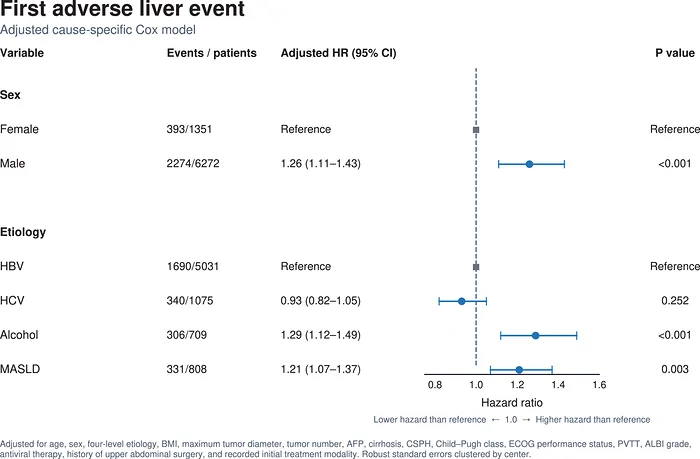
Adjusted cause‐specific HRs for the first adverse liver event by sex and etiology. Female sex and HBV are the reference categories; HRs greater than 1 indicate greater hazard. Covariates and center‐clustered robust standard errors are listed in the figure.

### 2.4 Subgroup and Interaction Analyses

Etiology‐stratified analyses showed lower mortality for women in the HBV group (HR, 0.89; 95% CI, 0.82–0.97; *p* = 0.007), whereas the HCV (HR, 0.94; 95% CI, 0.78–1.13; *p* = 0.506), alcohol (HR, 0.88; 95% CI, 0.71–1.09; *p* = 0.237), and MASLD (HR, 0.96; 95% CI, 0.79–1.17; *p* = 0.683) estimates were imprecise and not statistically significant (Figure S3). The male/female death counts were 2705/548 for HBV, 588/103 for HCV, 423/87 for alcohol, and 447/80 for MASLD (Table S11). Among men, survival differed across etiologies (*p* = 0.004); among women, the corresponding comparison was not statistically significant (*p* = 0.210; Figure S4). These within‐subgroup *p* values should not be interpreted as evidence of interaction.

The direction of the male‐versus‐female association with adverse liver events was generally similar across age, Barcelona Clinic Liver Cancer (BCLC) stage, Child–Pugh class, cirrhosis, recorded initial treatment modality, and China versus non‐China subgroups (Figure S5). Subgroup‐specific hazard ratios and confidence intervals were treated as descriptive; formal heterogeneity was assessed using interaction terms. For overall survival, effect modification by etiology was assessed using the joint test of the three sex‐by‐etiology product terms. The global 3‐df interaction test was not significant (*p* = 0.866; Table S14). In the sensitivity analysis excluding mixed etiologies, the corresponding global test remained non‐significant (*p* = 0.913; Table S5B).

## 3 Discussion

This large multicenter cohort provides real‐world evidence that female sex is associated with modestly better overall survival and fewer adverse liver events in HCC. The survival association persisted in matched and multivariable analyses that accounted for measured tumor, liver‐function, etiologic, and treatment factors. Alcohol‐related and MASLD‐related HCC were associated with greater liver‐event risk than HBV‐related HCC. For OS, the global sex‐by‐etiology interaction was not statistically significant, so differences in subgroup‐specific significance should not be interpreted as proven effect modification.

Sex differences in HCC incidence and outcomes have been described in population‐based and clinical cohorts, although estimates vary with age, surveillance, tumor stage, and treatment setting [4, 7, 13, 14, 15, 16]. Potential mechanisms include sex‐hormone signaling, IL‐6‐mediated inflammation, immune regulation, metabolic differences, exposure patterns, and treatment selection [8, 9, 17]. Our data extend prior observations by evaluating sex within a harmonized cohort spanning four major etiologic categories and a broad range of initial treatments. Nevertheless, the observed effect was modest and the retrospective design precludes causal interpretation; residual confounding remains possible despite matching and multivariable adjustment.

Etiology was more strongly associated with adverse liver events than with adjusted overall survival, consistent with evidence that HCC survival varies by underlying liver disease [12, 18]. Alcohol‐related and MASLD‐related liver disease may be accompanied by portal hypertension, metabolic comorbidity, and reduced hepatic reserve, which can increase vulnerability to decompensation [11, 19, 20, 21]. Beyond tumor‐ and liver‐related variables, host reserve, including nutritional status, may also influence long‐term outcomes after HCC treatment [22]. The component analysis and competing‐risk sensitivity provide a more clinically interpretable assessment than the composite estimate alone, because competing events can affect prognosis in cirrhosis [19]. Because the endpoint combines distinct outcomes, the component‐specific findings should be considered alongside the overall hazard ratios.

The HBV subgroup showed a statistically significant female survival association, whereas estimates in the smaller HCV, alcohol, and MASLD groups were not significant. The global interaction test, rather than differences in within‐subgroup significance, is the appropriate assessment of effect modification [23] and did not support heterogeneity by etiology. Wider confidence intervals in the non‐HBV groups indicate limited precision for modest subgroup effects. These results support cautious interpretation rather than a claim of an etiology‐independent biological advantage.

The study contributes in three main ways relative to prior registry‐based, molecular, or global observational studies [10, 13, 18, 24]: it includes viral, alcohol‐related, and metabolic etiologies in one clinical dataset; it evaluates overall survival together with adverse liver events classified locally using prespecified definitions and their components; and it incorporates multicenter treatment and liver‐function information that is often unavailable in administrative or genomic datasets. The multi‐country design broadens the clinical settings represented, but the cohort remains predominantly Chinese and should not be described as globally representative.

Several limitations merit emphasis. First, this was a retrospective observational study, and residual confounding, center‐specific measurement differences, and treatment‐selection bias cannot be eliminated; surveillance and stage at diagnosis may account for part of the apparent sex‐related survival difference. Because recorded initial treatment was ascertained after diagnosis and modeled as a fixed covariate, immortal‐time and treatment‐selection bias cannot be excluded, and treatment coefficients should not be interpreted causally. Second, 91.9% of patients were enrolled in China, which limits extrapolation to populations in which HCV, alcohol‐related liver disease, or MASLD predominate. Third, despite a prespecified hierarchy, mixed etiologies can be difficult to classify—particularly when metabolic dysfunction coexists with alcohol exposure—so misclassification remains possible. Fourth, detailed hormonal status, longitudinal disease activity, and some biological mediators were unavailable. Finally, adverse liver events form a heterogeneous composite; component events and causes of death were classified locally according to prespecified definitions rather than by a blinded central adjudication committee, and intercenter variation remains possible. Prospective studies with standardized central adjudication and broader geographic representation are needed. Because recruitment continued through February 2022 and follow‐up ended in March 2025, only early enrollees could contribute to the 60‐month risk set; the 5‐year survival estimates should therefore be interpreted as tail estimates with attention to the final number at risk.

## 4 Conclusion

Female sex was associated with modestly better overall survival and a lower hazard of adverse liver events after adjustment for measured clinical factors in this multicenter HCC cohort.

Alcohol‐related and MASLD‐related HCC were associated with greater liver‐event risk than HBV‐related HCC. The sex association with survival did not differ significantly by etiology, but subgroup precision and the China‐dominant cohort composition require cautious interpretation.

## 5 Materials and Methods

### 5.1 Study Design and Patients

This retrospective international multicenter cohort study consecutively included patients with newly diagnosed HCC from 41 tertiary referral centers in China, Brazil, Argentina, Italy, and Mexico between February 2020 and February 2022. All centers used a prespecified data dictionary and study protocol, and data‐quality queries were resolved before database locking. The study was conducted in accordance with the Declaration of Helsinki and was approved by the institutional review boards of participating centers, with a waiver of informed consent where permitted because of the retrospective design. Reporting was revised in accordance with STROBE principles.

Patients were eligible if they had (1) HCC confirmed histologically or by characteristic findings on contrast‐enhanced computed tomography or magnetic resonance imaging according to accepted international criteria; (2) newly diagnosed, treatment‐naive HCC at initial presentation; and (3) baseline clinical, laboratory, imaging, and outcome follow‐up data required for the prespecified analyses. The six mutually exclusive exclusion categories were another simultaneous malignancy, mixed hepatocellular‐cholangiocarcinoma, prior HCC‐directed antitumor treatment, recurrent HCC, ruptured HCC, and unavailable outcome follow‐up (Figure 1). After central data queries, all 7623 included patients had complete values for variables used in the primary models; therefore, no imputation was performed and the primary analyses used complete records.

The study size was determined by all eligible consecutive patients available during the prespecified recruitment period; no formal a priori sample‐size calculation was performed.

### 5.2 Etiology Classification

Underlying liver disease was represented by four mutually exclusive primary analytic categories: hepatitis B virus (HBV), hepatitis C virus (HCV), alcohol‐related liver disease, and metabolic dysfunction–associated steatotic liver disease (MASLD) [25]. These categories were prespecified by the source cohort, and all screened records contained the HBV, HCV, alcohol, and metabolic indicators needed to apply the classification rule; therefore, no included patient remained in an “other/unknown” category. Each etiologic criterion was recorded independently so that overlap could be identified before primary assignment.

HBV‐related HCC was defined by hepatitis B surface antigen positivity. HCV‐related HCC was defined by detectable HCV RNA or a documented history of chronic HCV infection with sustained virologic response. Alcohol‐related liver disease required documented average alcohol exposure of > 30 g/day in men or > 20 g/day in women for at least 5 years. MASLD‐related HCC required hepatic steatosis on imaging or histology plus at least one prespecified cardiometabolic criterion (overweight or obesity, dysglycemia or diabetes, hypertension, hypertriglyceridemia, or low HDL cholesterol). When biopsy material was available, histologic metabolic dysfunction–associated steatohepatitis (MASH) was defined by macrovesicular steatosis involving at least 5% of hepatocytes together with lobular inflammation and hepatocyte ballooning, with or without fibrosis. MASH was not inferred from imaging alone; non‐biopsy metabolic cases were classified as MASLD‐related HCC.

Patients meeting more than one etiologic definition were flagged as mixed etiology before primary assignment. The four‐level analytic assignment rule was applied deterministically: any HBV criterion assigned HBV; otherwise any HCV criterion assigned HCV; otherwise the alcohol criterion assigned alcohol‐related disease; and remaining patients meeting the metabolic criterion were assigned MASLD. This hierarchy was used only to create mutually exclusive analysis groups and was not interpreted as biological dominance. Overall, 486 patients (6.4%) met more than one definition; a sensitivity analysis excluded all mixed‐etiology cases. All included patients had a recorded primary category under this algorithm.

### 5.3 Clinical Variables and Definitions

Baseline variables included age, sex, body mass index, liver function, tumor characteristics, laboratory measures (including creatinine), comorbidities, antiviral therapy, smoking and alcohol histories, and history of upper abdominal surgery. Liver reserve was assessed using Child–Pugh class and albumin–bilirubin (ALBI) grade. Clinically significant portal hypertension (CSPH) was defined by gastroesophageal varices, ascites attributable to portal hypertension, or a hepatic venous pressure gradient of at least 10 mmHg when available. Sarcopenia was defined from a baseline computed‐tomography image at L3 obtained within 30 days of diagnosis; skeletal muscle was segmented semiautomatically from −29 to +150 HU, indexed to height squared, and classified using skeletal‐muscle‐index cutoffs < 42 cm^2^/m^2^ in men and < 38 cm^2^/m^2^ in women. Two radiologists reviewed segmentation, with discrepancies resolved by consensus.

Tumor burden was characterized by maximum tumor diameter, tumor number, alpha‐fetoprotein concentration, portal vein tumor thrombosis, and Barcelona Clinic Liver Cancer stage at diagnosis. Recorded initial treatment modality was defined as the first HCC‐directed treatment initiated within 30 days after diagnosis and was classified as surgical resection, curative local therapy (radiofrequency or microwave ablation), palliative locoregional therapy (transarterial chemoembolization [TACE]), systemic therapy (tyrosine kinase inhibitor [TKI] or immune checkpoint inhibitor [ICI]), or supportive care. For combination treatment, the prespecified hierarchy was resection, local therapy, palliative locoregional therapy (TACE), systemic therapy (TKI/ICI), and supportive care. Because treatment was recorded after time zero, treatment coefficients were used only for adjustment and were not interpreted causally.

### 5.4 Outcomes

Overall survival was the primary endpoint and was measured from the date of initial HCC diagnosis to death from any cause. Patients alive at the last documented contact were censored on that date.

The secondary composite endpoint, adverse liver events, was the time from HCC diagnosis to the first occurrence of hepatic decompensation, liver failure, or liver‐related death. Hepatic decompensation comprised new or worsening ascites requiring treatment or paracentesis, acute variceal hemorrhage, or overt hepatic encephalopathy (West Haven grade II or higher). Liver failure was defined as clinically documented acute or progressive hepatic failure with jaundice and/or coagulopathy accompanied by organ dysfunction. Liver‐related death was classified from source medical records as death directly attributable to liver failure, portal‐hypertensive complications, HCC‐related hepatic deterioration, or treatment‐related hepatic failure. Each patient contributed only one first‐event component; when multiple components occurred on the same date, the prespecified priority was liver‐related death, liver failure, then hepatic decompensation; within hepatic decompensation, the priority was encephalopathy, variceal bleeding, and then ascites. Deaths whose cause remained indeterminate after source review were not classified as liver‐related death. Component events and causes of death were classified locally using the common data dictionary; no blinded central adjudication committee was used. Liver transplantation was not counted as an adverse liver event.

### 5.5 Follow‐up

Patients were followed according to local clinical practice and international guidance. Follow‐up included clinical assessment, laboratory testing, and imaging at regular intervals. Survival status, component liver events, transplantation, and cause of death were ascertained from outpatient visits, hospital records, and structured follow‐up systems. The follow‐up cutoff was March 30, 2025. Potential follow‐up was summarized by the reverse Kaplan–Meier method; observed person‐time accrued from diagnosis to death or censoring, and numbers at risk were read directly from the Kaplan–Meier risk sets.

### 5.6 Statistical Analysis

Categorical variables were summarized as counts and percentages and compared using the chi‐square or Fisher exact test. Continuous variables were summarized as mean (standard deviation) or median (interquartile range), as appropriate, and compared using the Student's *t* test or Mann–Whitney *U* test. All reported percentages and continuous‐variable scales were rechecked against the table denominators.

A propensity score for female sex was estimated using age, body mass index, tumor size, tumor number, alpha‐fetoprotein, cirrhosis, CSPH, Child–Pugh class, ECOG performance status, etiology, portal vein tumor thrombosis, history of upper abdominal surgery, ALBI grade, ASA class, liver biochemistry, platelet count, creatinine, bilirubin, albumin, sarcopenia, smoking, alcohol use, antiviral therapy, comorbidities, and recorded initial treatment modality. Patients were matched 1:1 by nearest neighbor without replacement using a caliper of 0.1 standard deviations of the logit of the propensity score. The matched cohort comprised 1351 men and 1351 women. Balance was evaluated using absolute standardized mean differences (SMDs), with < 0.10 prespecified as acceptable; multicategory variables were summarized using a generalized SMD. The matched‐cohort Cox model used robust sandwich variance clustered by matched pair. For inverse‐probability weighting (IPW) targeting the average treatment effect (ATE), the same propensity‐score model was used to derive stabilized ATE weights, which were truncated at the first and 99th percentiles; robust sandwich standard errors accounted for clustering by center.

Overall survival was estimated by the Kaplan–Meier method and compared using the log‐rank test. Univariable and multivariable Cox proportional‐hazards models were fitted. Etiology entered the model as one four‐level categorical variable with HBV as the reference, and recorded initial treatment modality was included as a five‐level covariate. For the prespecified sex‐by‐etiology analysis, three product terms were entered and evaluated jointly using a 3‐degree‐of‐freedom Wald test. The proportional‐hazards assumption was assessed using Schoenfeld residuals. Primary full‐cohort standard errors were made robust to clustering within center; shared‐frailty and country‐stratified Cox models were treated as sensitivity analyses. Additional overall‐survival sensitivity analyses used IPW with robust sandwich standard errors, a Cox model stratified by matched pair, and a non‐China cohort analysis.

Time to first adverse liver event was analyzed using a cause‐specific Cox model adjusted for age, sex, etiology, body mass index, tumor size, tumor number, alpha‐fetoprotein, cirrhosis, CSPH, Child–Pugh class, ECOG status, portal vein tumor thrombosis, ALBI grade, antiviral therapy, history of upper abdominal surgery, and recorded initial treatment. In the cause‐specific analysis, patients were censored at non‐liver‐related or indeterminate death, liver transplantation, or last follow‐up. A complementary Fine–Gray model used the same adjustment set, treated non‐liver‐related or indeterminate death and liver transplantation as competing events, and used center‐clustered robust standard errors. Additional sensitivity analyses excluded mixed etiologies, fitted a center shared‐frailty model, and used an alternative Fine–Gray specification in which transplantation was censored and non‐liver‐related or indeterminate death was the competing event. The 90‐day landmark analysis was restricted to patients alive, uncensored, and adverse‐liver‐event‐free at day 90, with analysis time reset to day 90. Analyses used SPSS version 26.0 and R version 4.0.3; two‐sided *p* < 0.05 indicated statistical significance.

Potential selection bias was reduced through consecutive enrollment and prespecified eligibility criteria, confounding was addressed using propensity‐score matching and multivariable adjustment, and center heterogeneity was evaluated using cluster‐robust and sensitivity models.

## Author Contributions

Conceptualization and study design: Feng Xia, Hongwei Huang, Bin Guo, Maosen Xu, Rodrigo A. Gasque, Qian Chen, Qiao Zhang, Xiaoping Chen, Tianyu Geng, Fabio Giannone, Carlos Moctezuma‐Velazquez, Erlei Zhang, Wellington Andraus, Qi Cheng, and Ziqi Zhang. Methodology, formal analysis, visualization, and writing of the original draft: Feng Xia, Hongwei Huang, Bin Guo, Maosen Xu, Tianyu Geng, Qi Cheng, and Ziqi Zhang. Investigation, resources, data acquisition, data curation, and validation: Rodrigo A. Gasque, Qian Chen, Qiao Zhang, Zhenheng Wu, Wen Chen, Zhiyuan Huang, Yiyang Liu, Liping Liu, Shuangqin Han, Songqing He, Deyu Li, Bangde Xiang, Cuncai Zhou, Wei Zhou, Yuntong Li, Shounan Lu, Chenhao Jiang, Xibin Duan, Yubo Liao, Jian Gu, Bing Zhou, Wei Cai, Jiakai Zhang, Huaxuan Yin, Guobing Xia, Yunyan Wan, Jian Li, Xiafei Xia, Huapeng Sun, Shuai Wang, Yifa Chen, Fangjun Yuan, Fanfei Lin, Wenwen Cai, Hengyi Gao, and Li Ren. Supervision, project administration, and critical revision of the manuscript: Xiaoping Chen, Tianyu Geng, Fabio Giannone, Carlos Moctezuma‐Velazquez, Erlei Zhang, Wellington Andraus, Qi Cheng, and Ziqi Zhang. All authors contributed to interpretation of the findings and writing – review and editing. All authors have read and approved the final manuscript.

## Funding

This study was supported by the Joint Funds of the National Natural Science Foundation of Hubei (No. JCZRLH202500236), Postdoctor Research Fund of West China Hospital, Sichuan University (2024HXBH176) , National Natural Science Foundation of China (82303752) and the Joint Fund of the National Natural Science Foundation of China (No. U23A20483). The funders had no role in study design; data collection, analysis, or interpretation; manuscript preparation; or the decision to submit the manuscript for publication.

## Ethics Statement

Clinical study: This study was conducted in accordance with the Declaration of Helsinki and was approved by the Ethics Committee of West China Hospital (approval No. 2025‐8730). Participating centers obtained local ethics approval or an authorized waiver/reliance arrangement as required. The requirement for individual informed consent was waived where permitted because of the retrospective design and use of deidentified data. Animal experiments: Not applicable.

## Conflicts of Interest

The authors declare no conflicts of interest.

## Supporting information




**Supporting File**: mco271048‐sup‐0001‐SuppMat.docx.

## Data Availability

The deidentified participant‐level data are not publicly available because of patient privacy, multicenter data‐use agreements, and local ethics restrictions. The study data dictionary and analytic code may be made available to qualified researchers from the corresponding author upon reasonable request, subject to approval by the participating institutions and ethics committees and completion of any required data‐use agreement.
