# Supplementary material for: Sex and Etiology Differences in Overall Survival and Adverse Liver Events in Hepatocellular Carcinoma: An International Multicenter Study
Source: MedComm (2020). 2026 Nov 9;7(11):e71048. doi: 10.1002/mco2.71048 (PMC13748944; doi:10.1002/mco2.71048)
Supplement: Supplementary file 1 [file MCO2-7-e71048-s001.docx]

**Supplementary Information**

**Sex and Etiology Differences in Overall Survival and Adverse Liver Events in Hepatocellular Carcinoma: An International Multicenter Study**

Feng Xia^1,2#^, Hongwei Huang^2#^, Bin Guo^2#^, Maosen Xu^1#^, Rodrigo A. Gasque^3#^, Qian Chen^4#^, Qiao Zhang^5#^, Zhenheng Wu^6^, Wen Chen^7^, Zhiyuan Huang^8^, Yiyang Liu^9^, Liping Liu^10^, Shuangqin Han^11^, Songqing He^12^, Deyu Li^13^, Bangde Xiang^14^, Cuncai Zhou^15^, Wei Zhou^16^, Yuntong Li^17^, Shounan Lu^18^, Chenhao Jiang^19^, Xibin Duan^20^, Yubo Liao^21^, Jian Gu^22^, Bing Zhou^23^, Wei Cai^24^, Jiakai Zhang^25^, Huaxuan Yin^26^, Guobing Xia^27^, Yunyan Wan^28^, Jian Li^29^, Xiafei Xia^30^, Huapeng Sun^31^, Shuai Wang^32^, Yifa Chen^33^, Fangjun Yuan^34^, Fanfei Lin^35^, Wenwen Cai^36^, Hengyi Gao^37^, Li Ren^38^, Xiaoping Chen^2^, Tianyu Geng^39*^, Fabio Giannone^40*^, Carlos Moctezuma-Velazquez^41*^, Erlei Zhang^2*^, Wellington Andraus^42*^, Qi Cheng^2*^, Ziqi Zhang^1*^

1. Laboratory of Aging Research and Cancer Drug Target, State Key Laboratory of Biotherapy, National Clinical Research Center for Geriatrics, West China Hospital, Sichuan University, Chengdu, Sichuan, China.
2. Department of Hepatobiliary and Pancreatic Surgery, Tongji Hospital, Tongji Medical College of Huazhong University of Science and Technology, Wuhan, Hubei, China.
3. Hepatobiliary and Pancreatic Surgery Section, General Surgery Department, Clínica Universitaria Reina Fabiola, Facultad de Ciencias de la Salud, Universidad Católica de Córdoba, Córdoba, Argentina.
4. Department of Hepatobiliary Surgery, the First Affiliated Hospital of Shihezi University, Shihezi, Xinjiang, China.
5. Department of Hepatic Surgery, Zhongshan People's Hospital Affiliated to Guangdong Medical University, Zhongshan, Guangdong, China.
6. Department of Hepatopancreatobiliary Surgery, The First Affiliated Hospital of Fujian Medical University, Fuzhou, Fujian, China.
7. Department of Hepatobiliary Surgery, Fuzhou First General Hospital Affiliated of Fujian Medical University, Fuzhou, Fujian, China.
8. Department of General Surgery, General Hospital of Central Theater Command, Wuhan, Hubei, China.
9. Department of Hepatobiliary Surgery, Union Hospital, Tongji Medical College, Huazhong University of Science and Technology, Wuhan, Hubei, China.
10. Department of Hepatobiliary Surgery, Shenzhen People's Hospital, Shenzhen, Guangdong, China.
11. Department of Hepatobiliary Surgery, Tianjin First Central Hospital, Tianjin Medical University, Tianjin, China.
12. Department of Hepatobiliary Surgery, The First Affiliated Hospital of Guangxi Medical University, Guangxi Medical University, Nanning, Guangxi, China.
13. Department of Hepatobiliary Pancreatic Surgery, Henan Provincial People's Hospital, Zhengzhou University; Zhengzhou, Henan, China.
14. Department of Hepatobiliary, Pancreatic and Splenic Surgery, Guangxi Medical University Cancer Hospital, Guangxi Medical University, Nanning, Guangxi, China.
15. Department of Hepatobiliary Surgery, Jiangxi Cancer Hospital, Nanchang Medical College; Nanchang, Jiangxi, China.
16. Department of Hepatobiliary Surgery, Xiaogan Central Hospital, Xiaogan, Hubei, China.
17. Department of Hepatobiliary and Pancreatic Surgery, Zhongshan Hospital Xiamen University, Xiamen, Fujian, China.
18. Department of Minimally Invasive Liver Surgery, The First Affiliated Hospital of Harbin Medical University, Harbin, Heilongjiang, China.
19. Department of Hepatobiliary Surgery, The Second Affiliated Hospital of Army Medical University, Chongqing, China.
20. Department of Hepatobiliary and Pancreatic Surgery, Zhengzhou Central Hospital Affiliated to Zhengzhou University, Zhengzhou, Henan, China.
21. Department of Hepatobiliary and Pancreatic Surgery, The First Affiliated Hospital of Chengdu Medical College, Chengdu, Sichuan, China.
22. Department of Hepatobiliary Surgery, The First Affiliated Hospital of Nanjing Medical University, Nanjing, Jiangsu, China.
23. Department of General Surgery, The First Affiliated Hospital of Xinxiang Medical University, Xinxiang, Henan, China.
24. Department of Hepatobiliary Surgery, The First Affiliated Hospital of University of Science and Technology of China, Hefei, Anhui, China.
25. Department of Hepatobiliary and Pancreatic Surgery, The First Affiliated Hospital of Zhengzhou University, Zhengzhou, Henan, China.
26. Department of Hepatic-biliary-pancreatic Surgery, The First People's Hospital of Foshan, Foshan, Guangdong, China.
27. Department of Hepatobiliary and Pancreatic Surgery, Huangshi Central Hospital, Hubei Polytechnic University, Huangshi, Hubei, China.
28. Department of Hepatobiliary Pancreatic Surgery, Taihe Hospital, Shiyan, Hubei, China.
29. Department of Hepatobiliary Surgery, Renmin Hospital of Wuhan University, Wuhan, Hubei, China.
30. Department of Organ Transplantation, The First Affiliated Hospital of Kunming Medical University, Kunming Medical University, Kunming, Yunnan, China.
31. Department of General Surgery, Xiangyang Central Hospital, Affiliated Hospital of Hubei University of Arts and Science, Xiangyang, Hubei, China.
32. Department of Hepatobiliary Surgery, Jingzhou Central Hospital, Jingzhou, Hubei, China.
33. Department of Hepatobiliary Surgery, Ezhou Central Hospital, Ezhou, Hubei, China.
34. Department of General Surgery, Sinopharm Dongfeng General Hospital, Hubei University of Medicine, Shiyan, Hubei, China.
35. Department of Medicine, Fuzhou Second General Hospital, Fuzhou, Fujian, China.
36. Department of Emergency, Affiliated People’s Hospital of Jiangsu University, Zhenjiang, Jiangsu, China.
37. Department of Hepatobiliary and Pancreatic Surgery, Shenzhen Longhua District People's Hospital, Shenzhen, Guangdong, China.
38. Department of Hepatobiliary Surgery, Affiliated Hospital of Qinghai University, Xining, Qinghai, China.
39. College of Computer Science, Sichuan University, Chengdu, China.
40. Department of Hepato-Pancreato-Biliary, Oncologic and Robotic Surgery Unit, Azienda Ospedaliero-Universitaria SS. Antonio e Biagio e Cesare Arrigo, Alessandria, Italy.
41. Department of Gastroenterology, Instituto Nacional de Ciencias Médicas y Nutrición Salvador Zubirán, Mexico City, Mexico; Division of Gastroenterology (Liver Unit), University of Alberta, Edmonton, Canada.
42. Departamento de Gastroenterologia, Hospital das Clinicas HCFMUSP, Faculdade de Medicina, Universidade de Sao Paulo, Sao Paulo, SP, Brazil.

#These authors contributed equally to the article and should be listed as co-first authors.

*These authors are listed as co-corresponding authors.

**Corresponding authors:**

Ziqi Zhang, Laboratory of Aging Research and Cancer Drug Target, State Key Laboratory of Biotherapy, National Clinical Research Center for Geriatrics, West China Hospital, Sichuan University, Chengdu, Sichuan, China. (zhangziqicd@scu.edu.cn);

Qi Cheng, Department of Hepatobiliary and Pancreatic Surgery, Tongji Hospital, Tongji Medical College of Huazhong University of Science and Technology, 1095, Jiefang Avenue, Wuhan, China. (chengqi@hust.edu.cn);

Wellington Andraus, Departamento de Gastroenterologia, Hospital das Clinicas HCFMUSP, Faculdade de Medicina, Universidade de Sao Paulo, Sao Paulo, SP, Brazil. (wellingtonandraus@gmail.com);

Erlei Zhang, Department of Hepatobiliary and Pancreatic Surgery, Tongji Hospital, Tongji Medical College of Huazhong University of Science and Technology, 1095, Jiefang Avenue, Wuhan, China. (baiyu19861104@163.com);

Carlos Moctezuma-Velazquez, Department of Gastroenterology, Instituto Nacional de Ciencias Médicas y Nutrición Salvador Zubirán, Mexico City, Mexico; Division of Gastroenterology (Liver Unit), University of Alberta, Edmonton, Canada. (moctezum@ualberta.ca);

Fabio Giannone, Department of Hepato-Pancreato-Biliary, Oncologic and Robotic Surgery Unit, Azienda Ospedaliero-Universitaria SS. Antonio e Biagio e Cesare Arrigo, Alessandria, Italy. (giannone.cf@gmail.com);

Tianyu Geng, College of Computer Science, Sichuan University, Chengdu, China. (tygeng@scu.edu.cn).

**Supplementary Figure Legends**

Figure S1. Covariate balance before and after 1:1 propensity-score matching. Absolute standardized mean differences (SMDs) are shown before and after matching; the dashed line denotes the prespecified 0.10 threshold. Asterisks denote continuous covariates evaluated on the modeling scale, and multicategory covariates are summarized using one generalized SMD.

Figure S2. Sensitivity-analysis summary for overall survival and the first adverse liver event. Panel A shows female-versus-male HRs for overall survival; panel B shows male-versus-female HRs/sHRs for the first adverse liver event. The 90-day analysis was a landmark analysis restricted to patients alive, uncensored, and event-free at day 90, with time reset to day 90.

Figure S3. Overall survival stratified by sex within each prespecified etiologic group: (A) HBV, (B) HCV, (C) alcohol-related disease, and (D) MASLD. HRs compare female with male patients within each group.

Figure S4. Overall survival stratified by primary etiology within (A) male and (B) female patients. P values are from four-group log-rank tests.

Figure S5. Adjusted associations of male versus female sex with the first adverse liver event across prespecified subgroups. Subgroup-specific HRs and 95% CIs are descriptive; formal heterogeneity was assessed using interaction terms. The geographic comparison is China versus non-China; BCLC includes stages 0–C. Curative treatment comprises resection or local ablation; non-curative treatment comprises palliative locoregional therapy (TACE), systemic therapy (TKI/ICI), or supportive care.

**Figure S1.**

**Figure S2.**

**Figure S3.**

**Figure S4.**

**Figure S5.**

**Table S1. Baseline characteristics of included and excluded patients.**

|  |  | **Included** | **Excluded** | **P-value** |
| --- | --- | --- | --- | --- |
|  |  | **7623** | **821** |  |
| Sex (%) |  |  |  | 0.462 |
|  | Male | 6272 (82.3) | 667 (81.2) |  |
|  | Female | 1351 (17.7) | 154 (18.8) |  |
| Age (%) |  |  |  | 0.207 |
|  | < 60 y | 4611 (60.5) | 478 (58.2) |  |
|  | ≥ 60 y | 3012 (39.5) | 343 (41.8) |  |
| BMI (%) |  |  |  | 0.689 |
|  | < 25 kg/m2 | 5602 (73.5) | 598 (72.8) |  |
|  | ≥ 25 kg/m2 | 2021 (26.5) | 223 (27.2) |  |
| Tumor Size (%) |  |  |  | 0.527 |
|  | < 5 cm | 4155 (54.5) | 438 (53.3) |  |
|  | ≥ 5 cm | 3468 (45.5) | 383 (46.7) |  |
| Tumor Number (%) |  |  |  | 0.286 |
|  | 1 | 6975 (91.5) | 749 (91.2) |  |
|  | 2 | 445 (5.8) | 43 (5.2) |  |
|  | ≥ 3 | 203 (2.7) | 29 (3.5) |  |
| AFP (%) |  |  |  | 0.803 |
|  | < 400 ng/ml | 3089 (40.5) | 329 (40.1) |  |
|  | ≥ 400 ng/ml | 4534 (59.5) | 492 (59.9) |  |
| Cirrhosis (%) |  |  |  | 0.535 |
|  | No | 1565 (20.5) | 161 (19.6) |  |
|  | Yes | 6058 (79.5) | 660 (80.4) |  |
| CSPH (%) |  |  |  | 0.403 |
|  | No | 4977 (65.3) | 524 (63.8) |  |
|  | Yes | 2646 (34.7) | 297 (36.2) |  |
| Child–Pugh Class (%) |  |  |  | 0.891 |
|  | A | 5663 (74.3) | 604 (73.6) |  |
|  | B | 1258 (16.5) | 138 (16.8) |  |
|  | C | 702 (9.2) | 79 (9.6) |  |
| ECOG (%) |  |  |  | 0.689 |
|  | 0 | 4993 (65.5) | 532 (64.8) |  |
|  | ≥ 1 | 2630 (34.5) | 289 (35.2) |  |
| Etiology/Basic Liver Disease (%) |  |  |  | 0.278 |
|  | HBV | 5031 (66.0) | 532 (64.8) |  |
|  | HCV | 1075 (14.1) | 113 (13.8) |  |
|  | Alcohol | 709 (9.3) | 71 (8.6) |  |
|  | MASLD | 808 (10.6) | 105 (12.8) |  |
| PVTT (%) |  |  |  | 0.182 |
|  | No | 5832 (76.5) | 611 (74.4) |  |
|  | Yes | 1791 (23.5) | 210 (25.6) |  |
| History of upper abdominal surgery (%) |  |  |  | 0.217 |
|  | No | 7150 (93.8) | 761 (92.7) |  |
|  | Yes | 473 (6.2) | 60 (7.3) |  |
| ALBI Grade (%) |  |  |  | 0.293 |
|  | 1 | 6371 (83.6) | 676 (82.3) |  |
|  | 2 | 808 (10.6) | 86 (10.5) |  |
|  | 3 | 444 (5.8) | 59 (7.2) |  |
| ASA (%) |  |  |  | 0.355 |
|  | I | 1638 (21.5) | 170 (20.7) |  |
|  | II | 5153 (67.6) | 548 (66.7) |  |
|  | III | 832 (10.9) | 103 (12.5) |  |
| ALT (%) |  |  |  | 0.153 |
|  | < 50 U/L | 4863 (63.8) | 503 (61.3) |  |
|  | ≥ 50 U/L | 2760 (36.2) | 318 (38.7) |  |
| AST (%) |  |  |  | 0.397 |
|  | < 40 U/L | 3902 (51.2) | 433 (52.7) |  |
|  | ≥ 40 U/L | 3721 (48.8) | 388 (47.3) |  |
| ALP (%) |  |  |  | 0.108 |
|  | < 100 U/L | 5953 (78.1) | 621 (75.6) |  |
|  | ≥ 100 U/L | 1670 (21.9) | 200 (24.4) |  |
| GGT (%) |  |  |  | 0.178 |
|  | < 60 U/L | 5635 (73.9) | 589 (71.7) |  |
|  | ≥ 60 U/L | 1988 (26.1) | 232 (28.3) |  |
| Sarcopenia (%) |  |  |  | 0.395 |
|  | No | 3926 (51.5) | 410 (49.9) |  |
|  | Yes | 3697 (48.5) | 411 (50.1) |  |
| Smoking History (%) |  |  |  | 0.565 |
|  | No | 5452 (71.5) | 595 (72.5) |  |
|  | Yes | 2171 (28.5) | 226 (27.5) |  |
| Drinking History (%) |  |  |  | 0.180 |
|  | No | 6174 (81.0) | 649 (79.0) |  |
|  | Yes | 1449 (19.0) | 172 (21.0) |  |
| Antiviral therapy, n/N (%) |  |  |  | 0.619 |
|  | No | 2661 (34.9) | 276 (34.0) |  |
|  | Yes | 4962 (65.1) | 535 (66.0) |  |
|  | Missing | 0 (0.0) | 10 (1.2) |  |

The values in parentheses are percentages unless otherwise specified. Exclusion categories were mutually exclusive. Antiviral-therapy status was available for 811 of 821 excluded patients; 10 values were missing. Percentages and the P value for this variable use nonmissing observations.

**Table S2. Participating centers.**

| **Participating Center** | **City, Country** | **Region** |
| --- | --- | --- |
| Tongji Hospital of Huazhong University of Science and Technology | Wuhan, China | East Asia |
| Hospital das Clinicas HCFMUSP | Sao Paulo, Brazil | South America |
| West China Hospital | Chengdu, China | East Asia |
| The First Affiliated Hospital of Shihezi University | Shihezi, China | East Asia |
| The First Affiliated Hospital of Nanjing Medical University | Nanjing, China | East Asia |
| The First Affiliated Hospital of University of Science and Technology of China | Hefei, China | East Asia |
| Wuhan Union Hospital | Wuhan, China | East Asia |
| Clínica Universitaria Reina Fabiola | Córdoba, Argentina | South America |
| Zhongshan People's Hospital | Zhongshan, China | East Asia |
| The First Affiliated Hospital of Fujian Medical University | Fuzhou, China | East Asia |
| Fuzhou First General Hospital Affiliated of Fujian Medical University | Fuzhou, China | East Asia |
| General Hospital of Central Theater Command | Wuhan, China | East Asia |
| Shenzhen People's Hospital | Shenzhen, China | East Asia |
| Tianjin First Central Hospital | Tianjin, China | East Asia |
| The First Affiliated Hospital of Zhengzhou University | Zhengzhou, China | East Asia |
| The First Affiliated Hospital of Guangxi Medical University | Nanning, China | East Asia |
| Henan Provincial People's Hospital | Zhengzhou, China | East Asia |
| Azienda Ospedaliero-Universitaria SS. Antonio e Biagio e Cesare Arrigo | Alessandria, Italy | Europe |
| Guangxi Medical University Cancer Hospital | Nanning, China | East Asia |
| Jiangxi Cancer Hospital | Nanchang, China | East Asia |
| The First People's Hospital of Foshan | Foshan, China | East Asia |
| Huangshi Central Hospital | Huangshi, China | East Asia |
| Taihe Hospital | Shiyan, China | East Asia |
| Renmin Hospital of Wuhan University | Wuhan, China | East Asia |
| The First Affiliated Hospital of Kunming Medical University | Kunming, China | East Asia |
| Xiangyang Central Hospital | Xiangyang, China | East Asia |
| Jingzhou Central Hospital | Jingzhou, China | East Asia |
| Ezhou Central Hospital | Ezhou, China | East Asia |
| Sinopharm Dongfeng General Hospital | Shiyan, China | East Asia |
| Affiliated People’s Hospital of Jiangsu University | Zhenjiang, China | East Asia |
| Shenzhen Longhua District People's Hospital | Shenzhen, China | East Asia |
| Affiliated Hospital of Qinghai University | Xining, China | East Asia |
| The First Affiliated Hospital of Xinxiang Medical University | Xinxiang, China | East Asia |
| Zhongshan Hospital Xiamen University | Xiamen, China | East Asia |
| The First Affiliated Hospital of Harbin Medical University | Harbin, China | East Asia |
| The Second Affiliated Hospital of Army Medical University | Chongqing, China | East Asia |
| Zhengzhou Central Hospital Affiliated to Zhengzhou University | Zhengzhou, China | East Asia |
| The First Affiliated Hospital of Chengdu Medical College | Chengdu, China | East Asia |
| Instituto Nacional de Ciencias Médicas y Nutrición Salvador Zubirán | Mexico City, Mexico | North America |
| Fuzhou Second General Hospital | Fuzhou, China | East Asia |
| Xiaogan Central Hospital | Xiaogan, China | East Asia |

**Table S3. Distribution of centers and patients by country.**

| **Country** | **Centers, n** | **Patients, n (%)** |
| --- | --- | --- |
| China | 37 | 7,008 (91.9) |
| Brazil | 1 | 220 (2.9) |
| Argentina | 1 | 154 (2.0) |
| Italy | 1 | 136 (1.8) |
| Mexico | 1 | 105 (1.4) |
| Total | 41 | 7,623 (100.0) |

**Table S4. Selected baseline characteristics stratified by primary etiology.**

|  |  | **HBV** | **HCV** | **Alcohol** | **MASLD** | **P-value** |
| --- | --- | --- | --- | --- | --- | --- |
|  |  | **5031** | **1075** | **709** | **808** |  |
| Sex (%) |  |  |  |  |  | 0.038 |
|  | Male | 4103 (81.6) | 908 (84.5) | 578 (81.5) | 683 (84.5) |  |
|  | Female | 928 (18.4) | 167 (15.5) | 131 (18.5) | 125 (15.5) |  |
| Age (%) |  |  |  |  |  | 0.540 |
|  | < 60 y | 3019 (60.0) | 653 (60.7) | 433 (61.1) | 506 (62.6) |  |
|  | ≥ 60 y | 2012 (40.0) | 422 (39.3) | 276 (38.9) | 302 (37.4) |  |
| BMI (%) |  |  |  |  |  | 0.320 |
|  | < 25 kg/m2 | 3677 (73.1) | 785 (73.0) | 525 (74.0) | 615 (76.1) |  |
|  | ≥ 25 kg/m2 | 1354 (26.9) | 290 (27.0) | 184 (26.0) | 193 (23.9) |  |
| Tumor Size (%) |  |  |  |  |  | <0.001 |
|  | < 5 cm | 2607 (51.8) | 646 (60.1) | 386 (54.4) | 516 (63.9) |  |
|  | ≥ 5 cm | 2424 (48.2) | 429 (39.9) | 323 (45.6) | 292 (36.1) |  |
| Tumor Number (%) |  |  |  |  |  | <0.001 |
|  | 1 | 4678 (93.0) | 976 (90.8) | 573 (80.8) | 748 (92.6) |  |
|  | 2 | 227 (4.5) | 67 (6.2) | 113 (15.9) | 38 (4.7) |  |
|  | ≥ 3 | 126 (2.5) | 32 (3.0) | 23 (3.2) | 22 (2.7) |  |
| AFP (%) |  |  |  |  |  | <0.001 |
|  | < 400 ng/ml | 1809 (36.0) | 444 (41.3) | 293 (41.3) | 543 (67.2) |  |
|  | ≥ 400 ng/ml | 3222 (64.0) | 631 (58.7) | 416 (58.7) | 265 (32.8) |  |
| Cirrhosis (%) |  |  |  |  |  | <0.001 |
|  | No | 1099 (21.8) | 161 (15.0) | 113 (15.9) | 192 (23.8) |  |
|  | Yes | 3932 (78.2) | 914 (85.0) | 596 (84.1) | 616 (76.2) |  |
| CSPH (%) |  |  |  |  |  | <0.001 |
|  | No | 3376 (67.1) | 664 (61.8) | 448 (63.2) | 489 (60.5) |  |
|  | Yes | 1655 (32.9) | 411 (38.2) | 261 (36.8) | 319 (39.5) |  |
| Child–Pugh Class (%) |  |  |  |  |  | <0.001 |
|  | A | 3682 (73.2) | 794 (73.9) | 524 (73.9) | 663 (82.1) |  |
|  | B | 865 (17.2) | 178 (16.6) | 126 (17.8) | 89 (11.0) |  |
|  | C | 484 (9.6) | 103 (9.6) | 59 (8.3) | 56 (6.9) |  |
| ECOG (%) |  |  |  |  |  | 0.777 |
|  | 0 | 3276 (65.1) | 708 (65.9) | 473 (66.7) | 536 (66.3) |  |
|  | ≥ 1 | 1755 (34.9) | 367 (34.1) | 236 (33.3) | 272 (33.7) |  |
| PVTT (%) |  |  |  |  |  | 0.022 |
|  | No | 3888 (77.3) | 814 (75.7) | 545 (76.9) | 585 (72.4) |  |
|  | Yes | 1143 (22.7) | 261 (24.3) | 164 (23.1) | 223 (27.6) |  |
| History of upper abdominal surgery (%) |  |  |  |  |  | 0.797 |
|  | No | 4724 (93.9) | 1002 (93.2) | 668 (94.2) | 756 (93.6) |  |
|  | Yes | 307 (6.1) | 73 (6.8) | 41 (5.8) | 52 (6.4) |  |
| ALBI Grade (%) |  |  |  |  |  | <0.001 |
|  | 1 | 4236 (84.2) | 903 (84.0) | 589 (83.1) | 643 (79.6) |  |
|  | 2 | 553 (11.0) | 116 (10.8) | 78 (11.0) | 61 (7.5) |  |
|  | 3 | 242 (4.8) | 56 (5.2) | 42 (5.9) | 104 (12.9) |  |
| ASA (%) |  |  |  |  |  | 0.265 |
|  | I | 1072 (21.3) | 226 (21.0) | 155 (21.9) | 185 (22.9) |  |
|  | II | 3431 (68.2) | 721 (67.1) | 482 (68.0) | 519 (64.2) |  |
|  | III | 528 (10.5) | 128 (11.9) | 72 (10.2) | 104 (12.9) |  |
| ALT (%) |  |  |  |  |  | 0.200 |
|  | < 50 U/L | 3179 (63.2) | 687 (63.9) | 477 (67.3) | 520 (64.4) |  |
|  | ≥ 50 U/L | 1852 (36.8) | 388 (36.1) | 232 (32.7) | 288 (35.6) |  |
| AST (%) |  |  |  |  |  | 0.002 |
|  | < 40 U/L | 2611 (51.9) | 560 (52.1) | 369 (52.0) | 362 (44.8) |  |
|  | ≥ 40 U/L | 2420 (48.1) | 515 (47.9) | 340 (48.0) | 446 (55.2) |  |
| ALP (%) |  |  |  |  |  | 0.002 |
|  | < 100 U/L | 3964 (78.8) | 849 (79.0) | 551 (77.7) | 589 (72.9) |  |
|  | ≥ 100 U/L | 1067 (21.2) | 226 (21.0) | 158 (22.3) | 219 (27.1) |  |
| GGT (%) |  |  |  |  |  | 0.103 |
|  | < 60 U/L | 3697 (73.5) | 786 (73.1) | 526 (74.2) | 626 (77.5) |  |
|  | ≥ 60 U/L | 1334 (26.5) | 289 (26.9) | 183 (25.8) | 182 (22.5) |  |
| Sarcopenia (%) |  |  |  |  |  | 0.910 |
|  | No | 2576 (51.2) | 559 (52.0) | 369 (52.0) | 422 (52.2) |  |
|  | Yes | 2455 (48.8) | 516 (48.0) | 340 (48.0) | 386 (47.8) |  |
| Smoking History (%) |  |  |  |  |  | 0.735 |
|  | No | 3582 (71.2) | 774 (72.0) | 506 (71.4) | 590 (73.0) |  |
|  | Yes | 1449 (28.8) | 301 (28.0) | 203 (28.6) | 218 (27.0) |  |
| Comorbidities |  |  |  |  |  |  |
| Diabetes Mellitus (%) |  | 865 (17.2) | 196 (18.2) | 129 (18.2) | 160 (19.8) | 0.300 |
| Hypertension (%) |  | 1262 (25.1) | 278 (25.9) | 179 (25.2) | 222 (27.5) | 0.530 |
| Cardiovascular Disease (%) |  | 362 (7.2) | 84 (7.8) | 55 (7.8) | 71 (8.8) | 0.420 |
| Chronic Kidney Disease (%) |  | 281 (5.6) | 57 (5.3) | 40 (5.6) | 18 (2.2) | 0.001 |
| COPD (%) |  | 101 (2.0) | 24 (2.2) | 13 (1.8) | 11 (1.4) | 0.563 |

**Table S5A. Mixed-etiology classification.**

| **Mixed-etiology summary** | **Patients, n** |
| --- | --- |
| Patients meeting >1 etiologic definition | 486 (6.4% of 7,623) |
| Patients meeting one etiologic definition | 7,137 (93.6%) |
| Deterministic primary assignment rule | Any HBV → HBV; otherwise any HCV → HCV; otherwise alcohol → alcohol; remaining metabolic criterion → MASLD |
| Sensitivity cohort | Excluded all mixed-etiology cases |

**Table S5B. Sensitivity analyses after exclusion of mixed etiologies.**

| **Sensitivity analysis excluding mixed etiologies** | **Adjusted HR (95% CI)** | **P value** |
| --- | --- | --- |
| Overall survival: female vs male | 0.92 (0.85–0.99) | 0.033 |
| Sex × etiology (global 3-df test) | — | 0.913 |
| Adverse liver events: male vs female | 1.24 (1.09–1.42) | 0.001 |
| Adverse liver events: HCV vs HBV | 0.95 (0.83–1.09) | 0.461 |
| Adverse liver events: alcohol vs HBV | 1.27 (1.09–1.48) | 0.002 |
| Adverse liver events: MASLD vs HBV | 1.20 (1.05–1.38) | 0.009 |

**Table S6. Baseline characteristics stratified by sex before propensity-score matching.**

|  |  | **Male** | **Female** | **P-value** |
| --- | --- | --- | --- | --- |
|  |  | **6272** | **1351** |  |
| Age (%) |  |  |  | 0.113 |
|  | < 60 y | 3768 (60.1) | 843 (62.4) |  |
|  | ≥ 60 y | 2504 (39.9) | 508 (37.6) |  |
| BMI (%) |  |  |  | <0.001 |
|  | < 25 kg/m2 | 4541 (72.4) | 1061 (78.5) |  |
|  | ≥ 25 kg/m2 | 1731 (27.6) | 290 (21.5) |  |
| Tumor Size (%) |  |  |  | 0.049 |
|  | < 5 cm | 3,386 (54.0) | 769 (56.9) |  |
|  | ≥ 5 cm | 2,886 (46.0) | 582 (43.1) |  |
| Tumor Number (%) |  |  |  | 0.001 |
|  | 1 | 5762 (91.9) | 1213 (89.8) |  |
|  | 2 | 338 (5.4) | 107 (7.9) |  |
|  | ≥ 3 | 172 (2.7) | 31 (2.3) |  |
| AFP (%) |  |  |  | 0.286 |
|  | < 400 ng/ml | 2559 (40.8) | 530 (39.2) |  |
|  | ≥ 400 ng/ml | 3713 (59.2) | 821 (60.8) |  |
| Cirrhosis (%) |  |  |  | 0.001 |
|  | No | 1244 (19.8) | 321 (23.8) |  |
|  | Yes | 5028 (80.2) | 1030 (76.2) |  |
| CSPH (%) |  |  |  | 0.411 |
|  | No | 4108 (65.5) | 869 (64.3) |  |
|  | Yes | 2164 (34.5) | 482 (35.7) |  |
| Child–Pugh Class (%) |  |  |  | 0.021 |
|  | A | 4619 (73.6) | 1044 (77.3) |  |
|  | B | 1059 (16.9) | 199 (14.7) |  |
|  | C | 594 (9.5) | 108 (8.0) |  |
| ECOG (%) |  |  |  | 0.116 |
|  | 0 | 4133 (65.9) | 860 (63.7) |  |
|  | ≥ 1 | 2139 (34.1) | 491 (36.3) |  |
| Etiology/Basic Liver Disease (%) |  |  |  | 0.038 |
|  | HBV | 4103 (65.4) | 928 (68.7) |  |
|  | HCV | 908 (14.5) | 167 (12.4) |  |
|  | Alcohol | 578 (9.2) | 131 (9.7) |  |
|  | MASLD | 683 (10.9) | 125 (9.3) |  |
| PVTT (%) |  |  |  | 0.113 |
|  | No | 4776 (76.1) | 1056 (78.2) |  |
|  | Yes | 1496 (23.9) | 295 (21.8) |  |
| History of upper abdominal surgery (%) |  |  |  | 0.396 |
|  | No | 5876 (93.7) | 1274 (94.3) |  |
|  | Yes | 396 (6.3) | 77 (5.7) |  |
| ALBI Grade (%) |  |  |  | 0.040 |
|  | 1 | 5212 (83.1) | 1159 (85.8) |  |
|  | 2 | 689 (11.0) | 119 (8.8) |  |
|  | 3 | 371 (5.9) | 73 (5.4) |  |
| ASA (%) |  |  |  | 0.001 |
|  | I | 1398 (22.3) | 240 (17.8) |  |
|  | II | 4195 (66.9) | 958 (70.9) |  |
|  | III | 679 (10.8) | 153 (11.3) |  |
| ALT (%) |  |  |  | 0.006 |
|  | < 50 U/L | 4045 (64.5) | 818 (60.5) |  |
|  | ≥ 50 U/L | 2227 (35.5) | 533 (39.5) |  |
| AST (%) |  |  |  | 0.002 |
|  | < 40 U/L | 3261 (52.0) | 641 (47.4) |  |
|  | ≥ 40 U/L | 3011 (48.0) | 710 (52.6) |  |
| ALP (%) |  |  |  | 0.169 |
|  | < 100 U/L | 4879 (77.8) | 1074 (79.5) |  |
|  | ≥ 100 U/L | 1393 (22.2) | 277 (20.5) |  |
| GGT (%) |  |  |  | 0.002 |
|  | < 60 U/L | 4591 (73.2) | 1044 (77.3) |  |
|  | ≥ 60 U/L | 1681 (26.8) | 307 (22.7) |  |
| Platelets | ×10^9/L | 156 (117.5-239) | 161 (120-240) | 0.002 |
| Creatinine | mg/dL | 0.86 (0.79-0.99) | 0.89 (0.77-1.01) | 0.133 |
| Bilirubin | mg/dL | 0.66 (0.615-1.015) | 0.685 (0.58-0.99) | 0.276 |
| Albumin | g/L | 43.0 (38.0-47.0) | 44.0 (39.0-48.0) | 0.001 |
| Sarcopenia (%) |  |  |  | 0.428 |
|  | No | 3217 (51.3) | 709 (52.5) |  |
|  | Yes | 3055 (48.7) | 642 (47.5) |  |
| Smoking History (%) |  |  |  | <0.001 |
|  | No | 4329 (69.0) | 1123 (83.1) |  |
|  | Yes | 1943 (31.0) | 228 (16.9) |  |
| Drinking History (%) |  |  |  | <0.001 |
|  | No | 5033 (80.2) | 1,141 (84.5) |  |
|  | Yes | 1239 (19.8) | 210 (15.5) |  |
| Antiviral Therapy (%) |  |  |  | 0.222 |
|  | No | 2170 (34.6) | 491 (36.3) |  |
|  | Yes | 4102 (65.4) | 860 (63.7) |  |
| Comorbidities |  |  |  |  |
| Diabetes Mellitus (%) |  | 1098 (17.5) | 252 (18.7) | 0.317 |
| Hypertension (%) |  | 1574 (25.1) | 367 (27.2) | 0.113 |
| Cardiovascular Disease (%) |  | 457 (7.3) | 115 (8.5) | 0.121 |
| Chronic Kidney Disease (%) |  | 332 (5.3) | 64 (4.7) | 0.403 |
| COPD (%) |  | 113 (1.8) | 36 (2.7) | 0.038 |
| Treatment Modality |  |  |  | 0.021 |
| Surgical Resection (%) |  | 1951 (31.1) | 421 (31.2) |  |
| Curative Local Therapy (RFA/MWA) (%) |  | 666 (10.6) | 107 (7.9) |  |
| Palliative Locoregional Therapy (TACE) (%) |  | 2265 (36.1) | 517 (38.3) |  |
| Systemic Therapy (TKI/ICI) (%) |  | 1130 (18.0) | 238 (17.6) |  |
| Supportive Care (%) |  | 260 (4.1) | 68 (5.0) |  |

**Table S7. Baseline characteristics stratified by sex after 1:1 propensity-score matching.**

|  |  | **Male** | **Female** | **Absolute SMD†** |
| --- | --- | --- | --- | --- |
|  |  | **1351** | **1351** |  |
| Age (%) |  |  |  | 0.012 |
|  | < 60 y | 854 (63.2) | 843 (62.4) |  |
|  | ≥ 60 y | 497 (36.8) | 508 (37.6) |  |
| BMI (%) |  |  |  | 0.021 |
|  | < 25 kg/m2 | 1049 (77.6) | 1061 (78.5) |  |
|  | ≥ 25 kg/m2 | 302 (22.4) | 290 (21.5) |  |
| Tumor Size (%) |  |  |  | 0.026 |
|  | < 5 cm | 752 (55.7) | 769 (56.9) |  |
|  | ≥ 5 cm | 599 (44.3) | 582 (43.1) |  |
| Tumor Number (%) |  |  |  | 0.019 |
|  | 1 | 1205 (89.2) | 1213 (89.8) |  |
|  | 2 | 113 (8.4) | 107 (7.9) |  |
|  | ≥ 3 | 33 (2.4) | 31 (2.3) |  |
| AFP (%) |  |  |  | 0.023 |
|  | < 400 ng/ml | 515 (38.1) | 530 (39.2) |  |
|  | ≥ 400 ng/ml | 836 (61.9) | 821 (60.8) |  |
| Cirrhosis (%) |  |  |  | 0.009 |
|  | No | 326 (24.1) | 321 (23.8) |  |
|  | Yes | 1025 (75.9) | 1030 (76.2) |  |
| CSPH (%) |  |  |  | 0.014 |
|  | No | 860 (63.7) | 869 (64.3) |  |
|  | Yes | 491 (36.3) | 482 (35.7) |  |
| Child–Pugh Class (%) |  |  |  | 0.029 |
|  | A | 1028 (76.1) | 1044 (77.3) |  |
|  | B | 204 (15.1) | 199 (14.7) |  |
|  | C | 119 (8.8) | 108 (8.0) |  |
| ECOG (%) |  |  |  | 0.023 |
|  | 0 | 845 (62.5) | 860 (63.7) |  |
|  | ≥ 1 | 506 (37.5) | 491 (36.3) |  |
| Etiology/Basic Liver Disease (%) |  |  |  | 0.046 |
|  | HBV | 917 (67.9) | 928 (68.7) |  |
|  | HCV | 169 (12.5) | 167 (12.4) |  |
|  | Alcohol | 150 (11.1) | 131 (9.7) |  |
|  | MASLD | 115 (8.5) | 125 (9.3) |  |
| PVTT (%) |  |  |  | 0.021 |
|  | No | 1044 (77.3) | 1056 (78.2) |  |
|  | Yes | 307 (22.7) | 295 (21.8) |  |
| History of upper abdominal surgery (%) |  |  |  | 0.016 |
|  | No | 1269 (93.9) | 1274 (94.3) |  |
|  | Yes | 82 (6.1) | 77 (5.7) |  |
| ALBI Grade (%) |  |  |  | 0.044 |
|  | 1 | 1152 (85.3) | 1159 (85.8) |  |
|  | 2 | 112 (8.3) | 119 (8.8) |  |
|  | 3 | 87 (6.4) | 73 (5.4) |  |
| ASA (%) |  |  |  | 0.021 |
|  | I | 247 (18.3) | 240 (17.8) |  |
|  | II | 960 (71.1) | 958 (70.9) |  |
|  | III | 144 (10.7) | 153 (11.3) |  |
| ALT (%) |  |  |  | 0.011 |
|  | < 50 U/L | 811 (60.0) | 818 (60.5) |  |
|  | ≥ 50 U/L | 540 (40.0) | 533 (39.5) |  |
| AST (%) |  |  |  | 0.010 |
|  | < 40 U/L | 648 (48.0) | 641 (47.4) |  |
|  | ≥ 40 U/L | 703 (52.0) | 710 (52.6) |  |
| ALP (%) |  |  |  | 0.026 |
|  | < 100 U/L | 1088 (80.5) | 1074 (79.5) |  |
|  | ≥ 100 U/L | 263 (19.5) | 277 (20.5) |  |
| GGT (%) |  |  |  | 0.011 |
|  | < 60 U/L | 1050 (77.7) | 1044 (77.3) |  |
|  | ≥ 60 U/L | 301 (22.3) | 307 (22.7) |  |
| Platelets | ×10^9/L | 160 (123-247) | 161 (120-240) | 0.031 |
| Creatinine | mg/dL | 0.89 (0.77-1.01) | 0.89 (0.77-1.01) | 0.018 |
| Bilirubin | mg/dL | 0.68 (0.58-0.99) | 0.685 (0.58-0.99) | 0.027 |
| Albumin | g/L | 43.5 (38.5-47.5) | 44.0 (39.0-48.0) | 0.034 |
| Sarcopenia (%) |  |  |  | 0.006 |
|  | No | 705 (52.2) | 709 (52.5) |  |
|  | Yes | 646 (47.8) | 642 (47.5) |  |
| Smoking History (%) |  |  |  | 0.038 |
|  | No | 1142 (84.5) | 1123 (83.1) |  |
|  | Yes | 209 (15.5) | 228 (16.9) |  |
| Drinking History (%) |  |  |  | 0.022 |
|  | No | 1130 (83.6) | 1,141 (84.5) |  |
|  | Yes | 221 (16.4) | 210 (15.5) |  |
| Antiviral Therapy (%) |  |  |  | 0.006 |
|  | No | 487 (36.0) | 491 (36.3) |  |
|  | Yes | 864 (64.0) | 860 (63.7) |  |
| Comorbidities |  |  |  |  |
| Diabetes Mellitus (%) |  | 244 (18.1) | 252 (18.7) | 0.015 |
| Hypertension (%) |  | 362 (26.8) | 367 (27.2) | 0.008 |
| Cardiovascular Disease (%) |  | 118 (8.7) | 115 (8.5) | 0.008 |
| Chronic Kidney Disease (%) |  | 67 (5.0) | 64 (4.7) | 0.010 |
| COPD (%) |  | 33 (2.4) | 36 (2.7) | 0.014 |
| Treatment Modality |  |  |  | 0.011 |
| Surgical Resection (%) |  | 418 (30.9) | 421 (31.2) |  |
| Curative Local Therapy (RFA/MWA) (%) |  | 111 (8.2) | 107 (7.9) |  |
| Palliative Locoregional Therapy (TACE) (%) |  | 518 (38.3) | 517 (38.3) |  |
| Systemic Therapy (TKI/ICI) (%) |  | 233 (17.2) | 238 (17.6) |  |
| Supportive Care (%) |  | 71 (5.3) | 68 (5.0) |  |

† Absolute standardized mean differences (SMDs), rather than hypothesis-test P values, were used to assess balance after matching; <0.10 was prespecified as acceptable. Continuous covariates were evaluated on the model scale shown, and multicategory covariates were summarized using one generalized absolute SMD. Values correspond to Table S8 and Figure S1.

**Table S8. Absolute standardized mean differences before and after propensity-score matching.**

| **Covariate** | **Before matching** | **After matching** |
| --- | --- | --- |
| Age* | 0.047 | 0.012 |
| BMI* | 0.151 | 0.021 |
| Maximum tumor diameter* | 0.061 | 0.026 |
| log(AFP)* | 0.032 | 0.023 |
| log(ALT)* | 0.084 | 0.011 |
| log(AST)* | 0.094 | 0.010 |
| log(ALP)* | 0.035 | 0.026 |
| log(GGT)* | 0.098 | 0.011 |
| Platelets* | 0.059 | 0.031 |
| log(Creatinine)* | 0.025 | 0.018 |
| log(Bilirubin)* | 0.031 | 0.027 |
| Albumin* | 0.087 | 0.034 |
| Tumor number | 0.102 | 0.019 |
| Cirrhosis | 0.095 | 0.009 |
| CSPH | 0.025 | 0.014 |
| Child-Pugh class | 0.085 | 0.029 |
| ECOG | 0.047 | 0.023 |
| Etiology | 0.070 | 0.046 |
| PVTT | 0.048 | 0.021 |
| Upper abdominal surgery | 0.026 | 0.016 |
| ALBI grade | 0.074 | 0.044 |
| ASA class | 0.113 | 0.021 |
| Sarcopenia | 0.024 | 0.006 |
| Smoking history | 0.335 | 0.038 |
| Drinking history | 0.111 | 0.022 |
| Antiviral therapy | 0.037 | 0.006 |
| Diabetes | 0.030 | 0.015 |
| Hypertension | 0.047 | 0.008 |
| Cardiovascular disease | 0.046 | 0.008 |
| Chronic kidney disease | 0.026 | 0.010 |
| COPD | 0.058 | 0.014 |
| Initial treatment modality | 0.093 | 0.011 |

* Continuous covariates were evaluated on the modeling scale shown (log-transformed where indicated); multicategory covariates were summarized using one generalized absolute SMD.

**Table S9. Matched-cohort and weighting sensitivity analyses for overall survival.**

| **Model** | **Female vs male HR (95% CI)** | **P value** |
| --- | --- | --- |
| 1:1 PSM, robust SE clustered by matched pair | 0.91 (0.84–0.99) | 0.024 |
| IPW (ATE), stabilized weights and center-clustered robust SE† | 0.91 (0.85–0.98) | 0.008 |
| Cox model stratified by matched pair | 0.92 (0.85–1.00) | 0.045 |

† Stabilized average-treatment-effect (ATE) inverse-probability weights were estimated from the same propensity-score model as the matched analysis, truncated at the 1st and 99th percentiles, and entered in a weighted Cox model with robust sandwich standard errors clustered by center.

**Table S10. Sensitivity analyses accounting for center or country heterogeneity.**

| **Overall-survival sensitivity model** | **Female vs male HR (95% CI)** | **P value** |
| --- | --- | --- |
| Cox model with robust SE clustered by center | 0.91 (0.85–0.98) | 0.010 |
| Shared-frailty Cox model for center | 0.92 (0.86–0.99) | 0.023 |
| Cox model stratified by country | 0.91 (0.85–0.98) | 0.013 |
| Excluding 486 mixed-etiology cases | 0.92 (0.85–0.99) | 0.033 |
| Non-China cohort only (n = 615) | 0.94 (0.79–1.12) | 0.487 |

**Table S11. Sex-stratified sample sizes, deaths, Cox estimates, and precision by etiology.**

| **Etiology** | **Male, n / deaths** | **Female, n / deaths** | **Female vs male HR (95% CI)** | **P value** | **Precision note** |
| --- | --- | --- | --- | --- | --- |
| HBV | 4,103 / 2,705 | 928 / 548 | 0.89 (0.82–0.97) | 0.007 | Relatively precise |
| HCV | 908 / 588 | 167 / 103 | 0.94 (0.78–1.13) | 0.506 | Limited precision |
| Alcohol | 578 / 423 | 131 / 87 | 0.88 (0.71–1.09) | 0.237 | Limited precision |
| MASLD | 683 / 447 | 125 / 80 | 0.96 (0.79–1.17) | 0.683 | Limited precision |

The displayed subgroup estimates correspond to the reported Kaplan–Meier/Cox analyses; differences in within-subgroup significance were not interpreted as evidence of effect modification.

**Table S12A. Mutually exclusive first adverse liver-event and pre-event states.**

| **First adverse liver event** | **Female, n** | **Male, n** | **Total, n (%)** |
| --- | --- | --- | --- |
| Hepatic decompensation | 236 | 1,353 | 1,589 (20.8) |
| Ascites | 139 | 797 | 936 (12.3) |
| Variceal bleeding | 58 | 323 | 381 (5.0) |
| Hepatic encephalopathy | 39 | 233 | 272 (3.6) |
| Liver failure | 74 | 449 | 523 (6.9) |
| Liver-related death as the first adverse liver event | 83 | 472 | 555 (7.3) |
| Any first adverse liver event | 393 | 2,274 | 2,667 (35.0) |
| Death before a first adverse liver event (non-liver-related or indeterminate) | 436 | 1,976 | 2,412 (31.6) |
| Liver transplantation before a first adverse liver event | 14 | 60 | 74 (1.0) |
| No first adverse liver event, pre-event death, or pre-event transplantation by last follow-up | 508 | 1,962 | 2,470 (32.4) |
| Total | 1,351 | 6,272 | 7,623 (100.0) |

Each patient contributes once to the mutually exclusive first-state categories shown. Subsequent deaths after a nonfatal adverse liver event or transplantation are not counted again. Deaths of indeterminate cause occurring before any adverse liver event were competing deaths in the primary Fine–Gray analysis and were censored in the cause-specific Cox analysis.

**Table S12B. Adjusted Fine–Gray analysis of the first adverse liver event.**

| **Fine-Gray model** | **Adjusted sHR (95% CI)** | **P value** |
| --- | --- | --- |
| Male vs female | 1.23 (1.08–1.40) | 0.002 |
| HCV vs HBV | 0.95 (0.84–1.08) | 0.425 |
| Alcohol vs HBV | 1.25 (1.08–1.45) | 0.003 |
| MASLD vs HBV | 1.18 (1.04–1.34) | 0.010 |

Models used the covariate set specified for Table 3; non-liver-related or indeterminate death and liver transplantation were treated as competing events. Female sex and HBV were the reference categories.

**Table S13. Additional adverse-liver-event sensitivity analyses.**

| **Analysis** | **Male vs female HR/sHR (95% CI)** | **P value** |
| --- | --- | --- |
| Cause-specific Cox with center frailty | 1.25 (1.10–1.43) | <0.001 |
| Fine–Gray: transplantation censored; non-liver-related or indeterminate death competing | 1.24 (1.09–1.41) | 0.001 |
| 90-day landmark cause-specific Cox (n = 6,839; 2,229 post-landmark events): alive, uncensored, and event-free at day 90; time origin reset | 1.24 (1.09–1.42) | 0.001 † |

† The landmark analysis was restricted to patients alive, uncensored, and free of an adverse liver event at day 90; follow-up time was reset to day 90 and the Table 3 adjustment set was used.

**Table S14. Interaction between sex and etiology for overall survival.**

| **Effect-modification test** | **Degrees of freedom** | **P for interaction** |
| --- | --- | --- |
| Sex × etiology (global Wald test) | 3 | 0.866 |

Interaction was assessed using three sex-by-etiology product terms for the four-level nominal etiology factor and summarized by a joint 3-degree-of-freedom Wald test.
